# A Multi-layered Protein Network Stabilizes the *Escherichia coli* FtsZ-ring and Modulates Constriction Dynamics

**DOI:** 10.1371/journal.pgen.1005128

**Published:** 2015-04-07

**Authors:** Jackson Buss, Carla Coltharp, Gleb Shtengel, Xinxing Yang, Harald Hess, Jie Xiao

**Affiliations:** 1 Department of Biophysics and Biophysical Chemistry, Johns Hopkins University School of Medicine, Baltimore, Maryland, United States of America; 2 Janelia Farm Research Campus, Howard Hughes Medical Institute, Ashburn, Virginia, United States of America; Max Planck Institute for Terrestrial Microbiology, GERMANY

## Abstract

The prokaryotic tubulin homolog, FtsZ, forms a ring-like structure (FtsZ-ring) at midcell. The FtsZ-ring establishes the division plane and enables the assembly of the macromolecular division machinery (divisome). Although many molecular components of the divisome have been identified and their interactions extensively characterized, the spatial organization of these proteins within the divisome is unclear. Consequently, the physical mechanisms that drive divisome assembly, maintenance, and constriction remain elusive. Here we applied single-molecule based superresolution imaging, combined with genetic and biophysical investigations, to reveal the spatial organization of cellular structures formed by four important divisome proteins in *E. coli*: FtsZ, ZapA, ZapB and MatP. We show that these interacting proteins are arranged into a multi-layered protein network extending from the cell membrane to the chromosome, each with unique structural and dynamic properties. Further, we find that this protein network stabilizes the FtsZ-ring, and unexpectedly, slows down cell constriction, suggesting a new, unrecognized role for this network in bacterial cell division. Our results provide new insight into the structure and function of the divisome, and highlight the importance of coordinated cell constriction and chromosome segregation.

## Author Summary

Bacterial cell division is a highly regulated process that must be coordinated with other cellular processes (i.e. DNA replication and chromosome segregation) to promote faithful reproduction. In *Escherichia coli*, this regulation is most often mediated through the polymerization of the prokaryotic tubulin homolog, FtsZ, which forms a ring-like structure (FtsZ-ring) at midcell. The establishment of the FtsZ-ring marks the site of division and enables the assembly of the macromolecular division machinery (divisome). Here we applied single-molecule based superresolution imaging to reveal the three-dimensional structure of FtsZ in the context of its regulatory proteins: ZapA, ZapB and MatP. We found that these four proteins exist in a multi-layered network that extends from the cell membrane to the chromosome. This layered organization not only helps to stabilize the FtsZ-ring, but also serves to coordinate division with DNA status by influencing constriction rate. Our results not only provide a comprehensive view of the divisome, but also allow new insight to be garnered regarding the structure and function of the divisome.

## Introduction

Prokaryotic cell division is a conserved process that requires the formation of a multi-protein complex (divisome) at midcell [1]. Although many molecular constituents of the divisome have been identified [2], its *in-vivo* structural organization remains elusive. Understanding divisome architecture will help elucidate the mechanisms by which it achieves cytokinesis and coordinates with other cellular processes.

The central component of the divisome is FtsZ, a highly conserved prokaryotic tubulin homolog that polymerizes at midcell to form a ring-like structure [3]. The FtsZ-ring is not only required to serve as a stable scaffold for the assembly of all other division proteins [4], but may also generate a constrictive force during cytokinesis [5,6]. Based on *in vitro* polymerization studies, the basic structural units of the *E*. *coli* FtsZ-ring are believed to be single-stranded protofilaments that are on average 120 nm long [7]. These protofilaments are attached to the cytoplasmic membrane by binding to two membrane proteins, FtsA and ZipA [8,9].

Recently, high-resolution microscopy studies have revealed that the *in vivo* FtsZ-ring is a discontinuous structure, comprising a heterogeneous arrangement of FtsZ protofilaments [10–15]. Furthermore, FtsZ molecules within the ring have been shown to dynamically exchange with those in the cytoplasmic pool (half-time τ_1/2_ ≈ 10 – 30 s) [16,17]. These observations raise the question of how such a disordered, dynamic FtsZ-ring could provide the stable scaffold required for divisome assembly or generate a uniform constrictive force along the septum.

Recent work aimed at answering these questions have focused on a family of FtsZ-ring-associated proteins (Zaps), including: ZapA, ZapB, ZapC, ZapD and ZapE [18–23]. These proteins localize to the midcell in an FtsZ-dependent manner, and promote FtsZ-ring assembly *in vivo*. While no individual Zap is essential, single deletions lead to elongated cells, abnormal FtsZ-rings and irregular septum morphologies. Double and triple deletions result in synergistic defects [20,22]. These observations suggest that the Zaps carry out important, over-lapping roles in maintaining proper FtsZ-ring structure and function [20–23].

Among the five Zaps, ZapA and ZapB are best understood and are thought to work in concert to stabilize the FtsZ-ring [18,19,24]. We previously showed that in the absence of ZapA and/or ZapB, the FtsZ-ring dissociates into smaller, widely-dispersed FtsZ clusters throughout the midcell region, often leading to incomplete and abnormal septum formation [14]. These results support a model in which ZapA and ZapB stabilize the FtsZ-ring specifically at the division plane. *In vitro* characterization suggests that ZapA can stabilize the FtsZ-ring by cross-linking FtsZ protofilaments and possibly reducing its GTPase activity [24,25]. ZapB is a 100% coiled-coil protein that self polymerizes into large bundles *in vitro* [19]. ZapB does not interact directly with FtsZ, but associates indirectly through ZapA [26,27]. Hence, ZapB most likely exerts its stabilizing effect on FtsZ indirectly through ZapA. Interestingly, a recent confocal microscopy study observed that ZapB does not colocalize completely with the FtsZ-ring, but instead resides at the cytoplasmic face of the FtsZ-ring [26]. As such, the ZapB structure may have FtsZ-independent dynamics and functions, suggesting that the structural organization of the entire divisome is not simply dictated by the FtsZ-ring.

Further complicating the picture is the discovery of MatP, a DNA-binding protein involved in condensation and segregation of the terminus (*ter*) macrodomain of the chromosome [28,29]. MatP was found to interact with ZapB in a bacterial two-hybrid system [29] and recently shown to work with ZapB and ZapA to localize the divisome [30]. Thus, these data suggests a new, attractive model for the structural organization of the divisome—the divisome is extended from the membrane to the chromosome through a three-dimensional, extended protein network formed by these interacting proteins. This large protein network may provide the scaffold function previously attributed to FtsZ alone. Importantly, this network may also coordinate the progression of cell wall constriction with chromosome segregation. To test such a model, in this work we used quantitative superresolution imaging in conjunction with biophysical and genetic investigations to map the spatial organization and characterize the function of the three-dimensional cellular structures formed by FtsZ, ZapA, ZapB and MatP proteins in *E*. *coli* cells.

## Results

### Characterization of ZapA and ZapB structures in live *E*. *coli* cells

Using a single-molecule based superresolution imaging method, photoactivated-localization microscopy (PALM) [31], we first characterized the cellular structures formed by FtsZ, ZapA, or ZapB in live *E*. *coli* cells with a spatial resolution of ~45 nm [14]. We generated photoactivatable fluorescent fusion proteins mEos2-ZapA and ZapB-mEos2, and used an FtsZ-mEos2 construct described previously [10,14,32]. The mEos2-ZapA and ZapB-mEos2 fusions both rescued the elongated phenotypes of their respective deletion strains and localized to midcell in the absence of the endogenous protein (S1 Fig). To perform live-cell PALM imaging, we expressed these fusions ectopically in wild-type (wt) BW25113 cells and completed imaging in less than 30s. The estimated expression levels of FtsZ-mEos2, mEos2-ZapA and ZapB-mEos2 were ~30%, 45% and 10% of the total cellular concentration of the corresponding wt protein, respectively (Methods).

**Fig 1:**
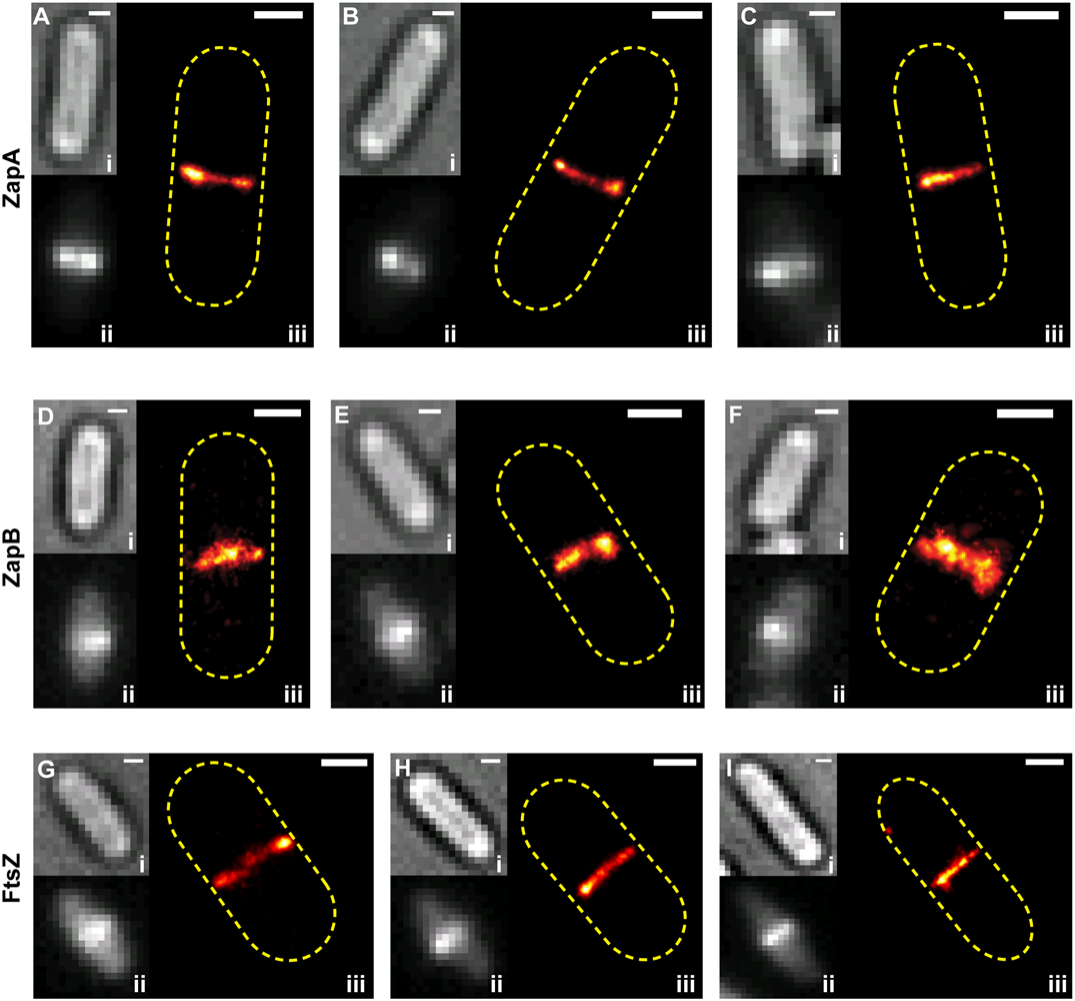
Live-cell PALM imaging of band-like ZapA, ZapB and FtsZ structures. Images of mEos2-ZapA (pJB051, A-C), ZapB-mEos2 (pJB045, D-F) and FtsZ-mEos2 (pJB042, G-I) in wt cells are shown in the order of bright-field image (i), ensemble fluorescence image (ii) and PALM image displayed in pseudocolor (iii). Approximate cell outlines are indicated by yellow dashed lines. Scale Bars, 500 nm.

We found that ZapA and ZapB predominantly formed band-like structures, indicative of rings projected to 2-dimensional (2D) imaging planes (Fig 1, S1 Table). The band-like structure was observed in 41% of cells expressing mEos2-ZapA (n_total_ = 229) and 59% of cells expressing ZapB-mEos2 (n_total_ = 137), similar to the prevalence observed for FtsZ-mEos2 (51%, n_total_ = 201). The remaining cells exhibited a number of different focal or non-planar morphologies, which were also observed previously for FtsZ [10,14] (S2 Fig). These alternate structures appear to precede the band-like structure as they were predominantly observed in shorter cells (S3 Fig, S1 Table). We observed similar morphologies when we applied superresolution imaging [33] to immuno-labeled, native FtsZ and ZapB structures, suggesting that the observed polymorphisms were not artifacts caused by the fused fluorescent proteins (S4 Fig).

While the three proteins displayed similar structural morphologies, we observed quantitative differences in the width (*w*) and diameter (*d*) of the band-like structures formed by each protein (Table 1, Methods). The mEos2-ZapA bands (*w* = 117 ± 4 nm, *d* = 686 ± 14 nm, n = 38; $\overset{-}{x}$ ± se) were similar (*p* > 0.3) to those of FtsZ-mEos2 (*w* = 115 ± 3 nm, *d* = 689 ± 11 nm, n = 54) (Table 1), but ZapB-mEos2 bands were significantly wider (161 ± 12 nm, *p* ≈ 1e^-4^, n = 34) and smaller in diameter (613 ± 16 nm, *p* < 5e^-5^). These structural differences are readily apparent in PALM images (Fig 1, iii), but obscured in the corresponding conventional fluorescence images (Fig 1, ii). The higher degree of structural similarity between ZapA and FtsZ relative to that of ZapB and FtsZ likely reflects the fact that ZapA binds FtsZ directly, while ZapB associates with FtsZ indirectly through ZapA [26,27].

**Table 1:** PALM measurements.

	n	Band Width (nm)	Diameter (nm)
FtsZ	54	115 ± 3	689 ± 11
ZapA	38	117 ± 4	686 ± 14
ZapB	34	161 ± 12	613 ± 16

### Two-color PALM imaging reveals structural deviations between FtsZ, ZapA and ZapB

Analysis of the single-color PALM images above indicated that ZapB structures could deviate from those of FtsZ and ZapA *in vivo*. Thus, to directly compare their spatial arrangements, we performed two-color PALM imaging in live *E*. *coli* cells. We expressed either Dronpa-ZapA or ZapB-Dronpa together with FtsZ-PAmCherry1 in the same cell. The functionality and expression level of these fusions were similar to those of the mEos2 constructs described above (S5 Fig).

**Fig 2:**
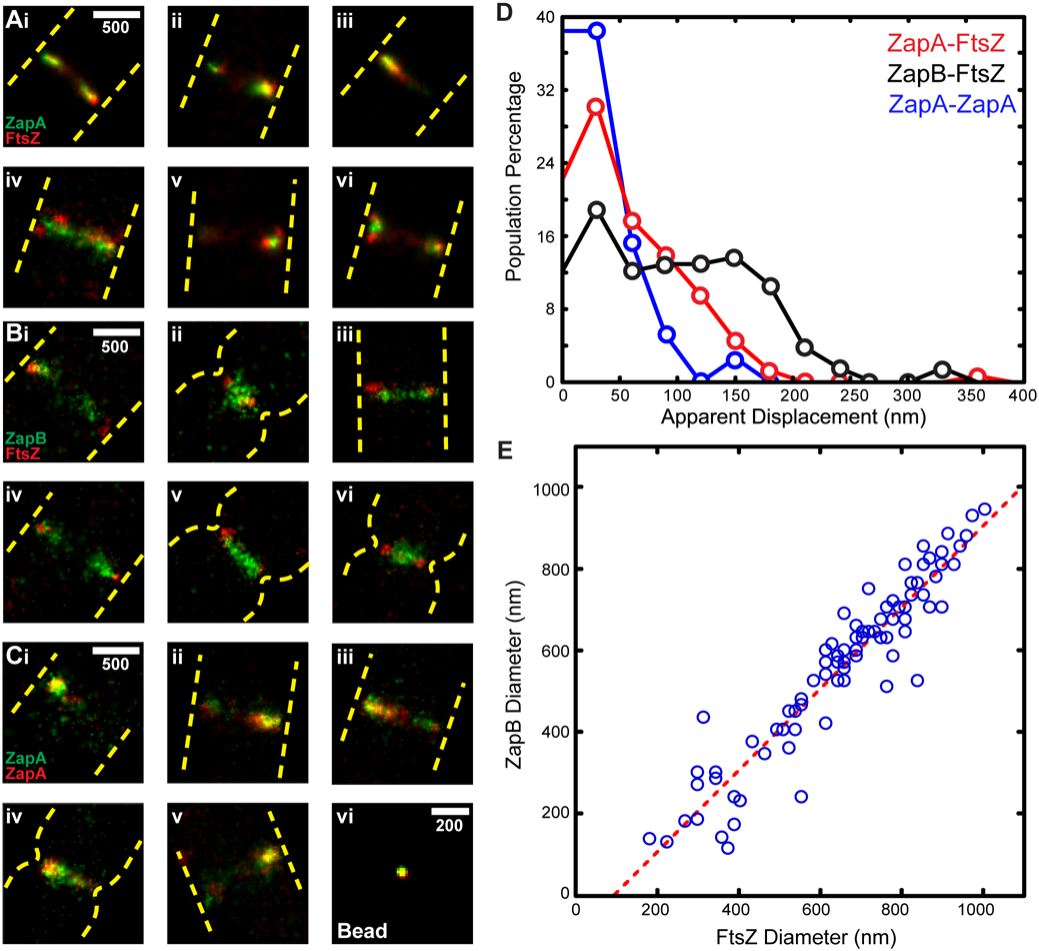
Two color-PALM imaging of ZapA-FtsZ, ZapB-FtsZ and ZapA-ZapA pairs. Cropped PALM images of cells expressing Dronpa-ZapA and FtsZ-PAmCherry1 (Ai-vi), ZapB-Dronpa and FtsZ-PAmCherry1 (Bi-vi), or Dronpa-ZapA and PAmCherry1-ZapA (Ci-v). Approximate cell outlines are indicated in yellow dashed lines. (Cvi) 2C-PALM image of a 100 nm TetraSpeck bead. (D) Histograms of the apparent displacement between the given protein pairs in individual cells. The mean values for the ZapA-FtsZ, ZapB-FtsZ and ZapA-ZapA pairs are 55 ± 50 nm (n = 158), 97 ± 70 nm (n = 132) and 33 ± 33 nm (n = 39), respectively ($\overset{-}{x}$ ± sd). (E) The diameters for ZapB-Dronpa and FtsZ-PAmCherry1 structures visualized simultaneously in the same cell (blue circles) were best fit to a line (red) where y = 1.0x - 95. The R^2^ of the fitted line to the data was 0.89. Scale Bars, as labeled in nm.

We found that structures formed by Dronpa-ZapA and FtsZ-PAmCherry1 largely overlapped, and adopted similar structural morphologies in ~80% of cells, consistent with incorporation of ZapA into the FtsZ-ring (Fig 2Ai-iii, n_total_ = 96). In the remaining cells, however, the two structures showed deviations significantly larger than our spatial resolution in two-color PALM imaging (~50 nm) (Fig 2Aiv-vi). On several occasions (n = 7), Dronpa-ZapA structures were sandwiched in between FtsZ-PAmCherry1 structures (Fig 2Av-vi), suggesting a possible bridging function for ZapA. We observed more substantial deviations between ZapB-Dronpa and FtsZ-PAmCherry1 (Fig 2B). In ~70% of cells (n_total_ = 88), ZapB-Dronpa structures appeared to be encompassed by FtsZ-PAmCherry1 and located toward the inner surface of FtsZ-ring (Fig 2B).

The structural deviations we observed between the FtsZ-ZapA and FtsZ-ZapB pairs were not observed in a control strain where Dronpa-ZapA was co-expressed with PAmCherry1-ZapA and imaged under the same condition (Fig 2Ci-v). Multi-color fluorescent beads imaged in both color channels also showed complete overlap within our spatial resolution (Fig 2Cvi). These results suggest that the observed structural deviations were not imaging artifacts, nor caused by the fusion protein dynamics or photoproperties of Dronpa and PAmCherry1.

To quantify the degree of colocalization for the ZapA-FtsZ, ZapB-FtsZ and ZapA-ZapA pairs, we performed a coordinate-based cross-correlation analysis [34–36]. In each cell, we determined the cross-correlation value between two different species at midcell as a function of their displacement along the short axis of the cell. The displacement value at maximal cross-correlation value was assigned as the 'apparent displacement' for each cell (Fig 2D). We found that the average apparent displacement between Dronpa-ZapA and FtsZ-PAmCherry1 molecules was 55 ± 50 nm (n = 158; $\overset{-}{x}$ ± sd). Molecules of ZapB-Dronpa were displaced farther away from FtsZ-PAmCherry1 molecules with an average of 97 ± 70 nm (n = 132). Both of these displacements are significantly larger than that of the control ZapA-ZapA pair (33 ± 33 nm, n = 39) (Fig 2D). The latter reflects our spatial resolution in resolving two cellular structures. These results further support our qualitative observations that ZapA can appreciably deviate from the FtsZ-ring, and that ZapB assembles into a discrete structure internal to the FtsZ-ring.

### Separation between FtsZ and ZapB ring-like structures remains constant throughout constriction

In cells showing visible constrictions, we often observed that both FtsZ and ZapB formed ring-like structures. To investigate whether the relative spatial arrangement between FtsZ and ZapB changes during cell constriction, we measured the corresponding diameters of these ring-like structures in each individual cell. We found that the diameters of the FtsZ-ring and ZapB-rings were linearly correlated with each other, and the scatter plot can be best fit by a line with a slope of 1.0 and x-intercept at 95 nm (Fig 2E). This observation indicates that the radial separation (95/2 = 47.5 nm) between FtsZ and ZapB structures is maintained throughout constriction. These results suggest that there may be a specific mechanism to maintain the relative spatial separation between FtsZ and ZapB, and such a separation may be important for successful cell wall constriction.

### iPALM reveals relative spatial arrangements of FtsZ, ZapA and ZapB with respect to the inner membrane

While two-color PALM imaging allowed us to directly visualize the relative arrangement between two protein species in the same cell, the nature of 2D imaging of three-dimensional (3D) structures prevented us from accurately quantifying the relative spatial arrangement of FtsZ, ZapA and ZapB along the radial axis. Therefore, we performed 3D superresolution imaging using interferometric PALM (iPALM). iPALM employs the same principles as PALM imaging, but also utilizes the interference of light from the same molecule in two different paths to determine its *z*-position with high precision [37,38]. Under our imaging conditions we achieved a, *x*-, *y*-, and *z*-resolution of ~20 nm.

**Fig 3:**
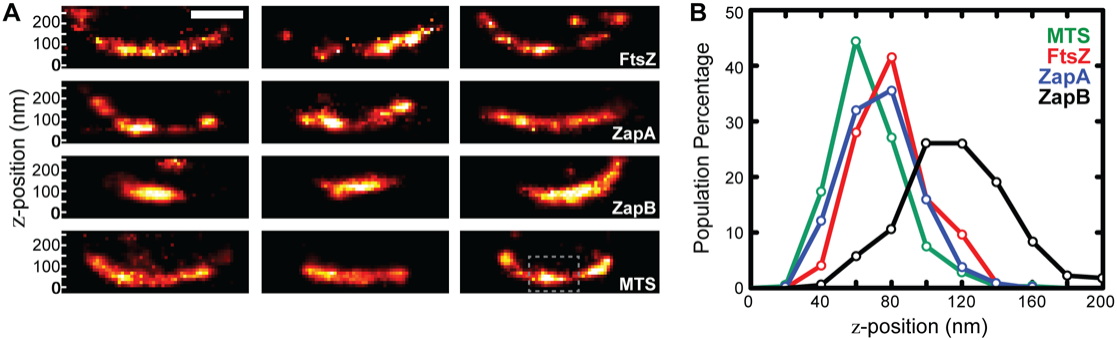
iPALM imaging and *z*-position measurements of FtsZ, ZapA, and ZapB. (A) Cross-sectional views of the midcell region of representative wt cells expressing FtsZ-mEos2, mEos2-ZapA, ZapB-mEos2 or mEos2-MTS_Bs_ (three cells for each strain) are displayed in respective rows. All images are to scale and share the same *z*-dimensions as labeled on the left. The dashed rectangle box (gray) in the bottom right-most image is representative of the user-defined box we used to calculate mean *z*-positions. (B) Histograms (20 nm bins) of the average measured *z*-positions for each protein structure in individual cells illustrate the internal nature of ZapB. Scale Bar, 250 nm.

iPALM images of FtsZ-mEos2 and mEos2-ZapA showed similar, irregular punctate structures that curved along the cell periphery (Fig 3A). ZapB-mEos2, however, appeared much more cohesive, exhibiting large, contiguous structures that often lacked curvature. This observation is consistent with our previous observations that ZapB structures significantly deviate from FtsZ and ZapA.

To measure the relative radial displacement of FtsZ, ZapA and ZapB molecules, we compared their mean *z*-positions to that of the coverslip surface coated with Alexa Fluor 568 [38]. To restrict our analysis to midcell regions close to the coverslip, we excluded cells that showed visible indentation at midcell in corresponding DIC (Differential Interference Contrast) images, and only used molecules inside a user-defined region at the bottom of each non-constricting cell (Fig 3A). The mean *z*-position of these molecules was then calculated for each individual cell, and the corresponding distribution for each protein in different cells was plotted in Fig 3B. The distributions of the three proteins show substantial overlap, but clearly indicate that ZapB molecules are displaced internal to the FtsZ-ring. Quantifying the mean *z*-positions reveals that FtsZ and ZapA structures reside close to each other (*z* = 80 ± 2 nm, n = 125 for FtsZ, and 74 ± 1 nm, n = 250 for ZapA, respectively; $\overset{-}{x}$ ± se), while the ZapB structures are displaced significantly internal to the FtsZ-ring (*z* = 117 ± 2 nm, n = 226). We note that the ~40 nm difference between FtsZ and ZapB observed by iPALM is in a similar range with what can be estimated from single-color, 2D PALM measurements (343_FtsZ radius_ – 306_ZapB radius_ = 37 nm), or from the correlation between the diameters of ZapB and FtsZ band-like structures in two-color, 2D PALM imaging (x-intercept / 2 = 47.5 nm, Fig 2E).

To orient these structures in the context of the inner membrane (IM), we determined the *z*-position of mEos2 fused to the membrane-targeting sequence of MinD (V^199^-S^219^) from *Bacillus subtilis* (mEos2-MTS_*Bs*_) (Fig 3A). mEos2-MTS_Bs_ distributed randomly and sparsely on the membrane; the mean *z*-position from the coverslip surface was measured at z = 67 ± 1 nm (n = 315), consistent with the expected value when the thickness of cell envelope was considered [39,40]. Thus, we used the z-position of mEos2-MTS_Bs_ as a proxy for the cytoplasmic face of the inner membrane and estimated that FtsZ is displaced ~13 nm away from the IM on average.

We note that our estimates of apparent displacement do not take into account the molecular size of the mEos2 label (~4 nm) [41]. To determine the true displacement between two mEos2-labeled proteins, one must know how mEos2 is oriented with respect to each labeled protein. Without this information, however, we can still estimate the error associated with our apparent displacement measurements by treating the size of mEos2 as an uncertainty ($\sqrt{4^{2}+4^{2}}=\text{5}.7 nm$). Comparing this uncertainty to the measured apparent displacements of FtsZ-ZapA (6 nm), FtsZ-ZapB (37 nm) and FtsZ-IM (13 nm), we conclude that FtsZ and ZapA reside on a similar radial plane, and that ZapB is significantly displaced into the cytoplasm relative to FtsZ. The 13 nm distance between FtsZ and the IM requires more careful consideration because the 5.7 nm uncertainty level is relatively large. Nevertheless, by taking into account the size of mEos2 and how mEos2 is attached to FtsZ, the displacement between FtsZ and the IM measured from iPALM can be estimated (S1 Text). This estimate will allow us to assess the amount of force FtsZ protofilaments could exert on the membrane through specific force generation mechanisms.

### MatP enhances FtsZ-ring stability

Next, we investigated the role of MatP in maintaining the layered FtsZ-ZapA-ZapB structure. We previously showed that deletion of MatP resulted in dispersed, mislocalized FtsZ structures under fast growth conditions [14]. This effect can be explained by an indirect mechanism, in which the abnormal nucleoid architecture caused by deletion of *matP* [28] results in aberrant distributions of the nucleoid-occlusion factor SlmA [42], which consequently disrupts FtsZ-ring assembly at the midcell. Our previous observation of SlmA mislocalization in Δ*matP* cells supported this mechanism [14]. To further test whether MatP also has a direct role in maintianing FtsZ-ring at the midcell through its interaction with ZapB and ZapA, we constructed a double deletion strain (Δ*matP*Δ*slmA*), which allowed us to observe the effect of Δ*matP* independent of the nucleoid-occlusion effect caused by SlmA. We reasoned that if abnormally-distributed SlmA is solely responsible for mislocalizing the FtsZ-ring in Δ*matP* cells, then deletion of *slmA* should revert cells to normal FtsZ-ring localization, as deletion of *slmA* alone does not show any detectable defect in FtsZ-ring localization (S6 Fig) [42]. In contrast to this expectation, we found that Δ*matP*Δ*slmA* cells displayed a similar FtsZ-ring mislocalization phenotype as Δ*matP*, albeit with a slightly reduced frequency (S6 Fig). This observation indicates that MatP has a SlmA-independent role in stabilizing the FtsZ-ring at midcell, possibly mediated by the physical connection between FtsZ and MatP through ZapA and ZapB. This is consistent with a recent report in which similar FtsZ-ring positioning defect was observed in related mutant backgrounds [30].

### MatP is located ~30 nm internal to ZapB

**Fig 4:**
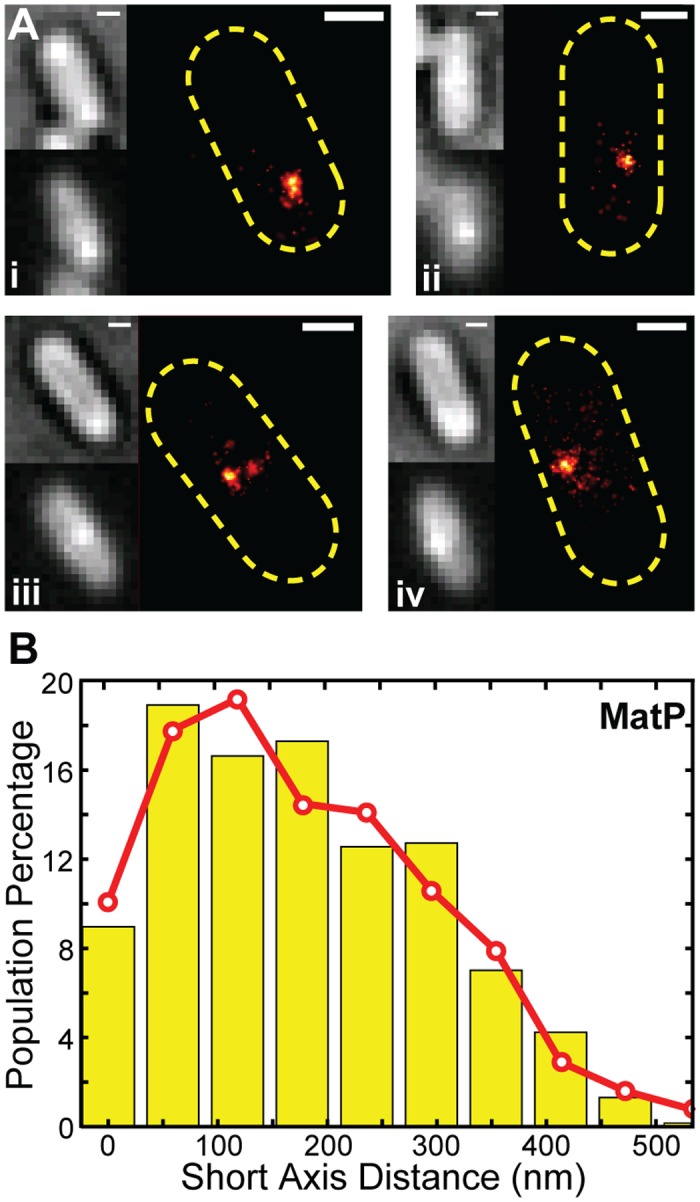
PALM imaging of MatP and *z*-position estimation. (A) Four representative PALM images for cells expressing MatP-mEos2 in the order of bright-field, ensemble fluorescence, and PALM image. Scale Bars, 500 nm. (B) Distribution of short-axis displacements of MatP-mEos2 clusters from the middle of the cell (yellow bars) fitted with a model (red line) in which a normally-distributed radial displacement is randomly projected to a 2D imaging plane (S1 Text). The fitted radial displacement is 285 ± 120 nm (red line, $\overset{-}{x}$ ± sd).

To determine where MatP is positioned inside the cell with respect to ZapB, we performed live-cell PALM imaging on wt and Δ*matP* cells ectopically expressing a MatP-mEos2 fusion protein (Fig 4Ai-iv). Consistent with previous studies [28,29], MatP-mEos2 typically appeared as one or two large clusters in both strains, suggesting that MatP-mEos2 localizes correctly. The MatP-mEos2 clusters we observed were on average 100 ± 58 nm in diameter ($\overset{-}{x}$ ± sd, n = 613, S7 Fig). Given our resolution, this size is much larger than a single molecule of MatP-mEos2 would appear, suggesting that the clusters likely comprise multiple closely-associated MatP molecules. This finding is consistent with the ability of MatP dimers to associate with a series of *matS* sites on the chromosome and further tetramerize to condense the *ter* macrodomain [43].

**Fig 5:**
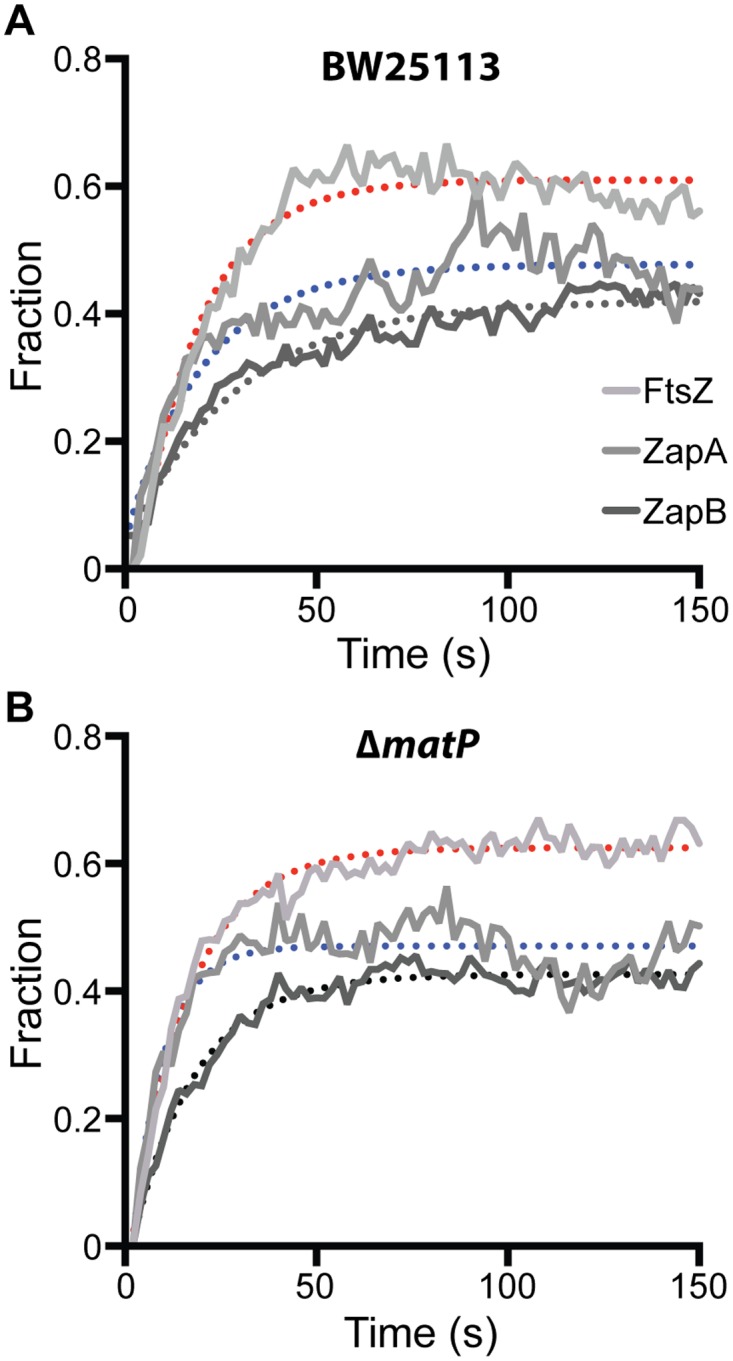
FRAP measurements of FtsZ, ZapA and ZapB turnover rates in BW25113 and Δ*matP* cells. Average fluorescence recovery trajectories for FtsZ (light grey), ZapA (grey) and ZapB (dark grey) are displayed for wt (A) and Δ*matP* (B) cells as a fraction of pre-bleached intensity. Trajectories were fit with single exponentials illustrated as dotted lines (FtsZ, red; ZapA, blue; ZapB, black). The respective half-times are listed in Table 2.

We measured the displacement of each MatP-mEos2 cluster from the cell center along the long and short axes of the cell (S8A Fig). We found that the long axis displacement exhibited a cell-length dependent decrease (S8B Fig), as expected from the directed movement of the *ter* macrodomain toward midcell during the cell cycle [28]. The short axis displacement, however, was independent of cell length (S8C Fig). The short axis displacement is the 2D projection of the radial displacement of each cluster from the cell center (S8A Fig). Assuming that MatP-mEos2 clusters are randomly distributed angularly and projected to the 2D imaging plane, the observed short axis displacement distribution of MatP-mEos2 clusters can be best described by a model in which MatP clusters are on average 280 ± 120 nm ($\overset{-}{x}$ ± sd, n = 613) radially away from the cell center (S1 Text, Figs 5B, S8–S9). Comparing this measurement with the typical diameter of ZapB band-like structures, we estimate that MatP resides ~30 nm (607_ZapB radius_ ÷ 2–280) internal to ZapB on average. Thus, MatP forms another distinct layer internal to the FtsZ-ring. Given the wide distributions of MatP and ZapB z-positions (Fig 3B, 4B), our data suggest that MatP and ZapB are appropriately positioned to enable direct interaction, and that this interaction serves to stabilize the FtsZ-ring. We note, however, that we cannot exclude the possibility that other proteins may bridge the ZapB-MatP interaction.

### Deletion of *matP* increases the turnover rates of FtsZ, ZapA and ZapB

As we have shown, MatP plays a direct role in stabilizing the FtsZ-ring through its interaction with ZapB. To further understand how MatP exerts this influence, we probed the turnover dynamics of FtsZ, ZapA, and ZapB in the presence and absence of MatP. Previous fluorescence recovery after photobleaching (FRAP) measurements showed that the FtsZ-ring and its associated proteins exchange dynamically with their cytoplasmic pools with a half-time of 10–30 s depending on growth conditions and strain background [16,17,25,44]. We reason that one way for MatP to stabilize the FtsZ-ring could be by promoting the stable formation of large, polymerized ZapB assemblies at midcell, which consequently stabilizes ZapA and FtsZ, and could be reflected by a reduction in the turnover rate of ZapB.

We ectopically expressed FtsZ-GFP, GFP-ZapA or GFP-ZapB in wt and Δ*matP* cells, and used FRAP to measure their turnover half-time under our slow growth conditions. Interestingly, we found that in the wt background, the FRAP half-time for FtsZ-GFP and GFP-ZapA were comparable to each other at 12.3 ± 0.2 (n = 58, $\overset{-}{x}$ ± se) and 14.2 ± 1.1 s (n = 51), respectively, but that of GFP-ZapB was significantly longer (19.8 ± 1.0 s, n = 59, p < 1e-10) (Fig 5A, Table 2). The slower turnover rate of GFP-ZapB is consistent with the highly polymeric nature of ZapB assemblies observed *in vitro* [19,27], and also agrees with our *in vivo* observations that ZapB structures are generally larger and more cohesive compared to those of FtsZ and ZapA (Fig 3A). In the Δ*matP* mutant, the turnover half-times of both GFP-ZapB and GFP-ZapA were significantly reduced by ~50% to 11.6 ± 0.4 s (n = 56, p < 0.001) and 5.7 ± 0.2 s (n = 59, p< 0.001), respectively, supporting an influential role for MatP on their turnover dynamics (Fig 5B, Table 2). The half-time of FtsZ-GFP turnover was also significantly reduced in the Δ*matP* mutant (10.5 ± 0.3 s, n = 59, p< 0.001), but to a lesser degree (~15%). This smaller effect may be due to the fact that MatP’s interaction with FtsZ is more indirect than its interactions with ZapA or ZapB, and/or that FtsZ’s self-polymerization properties and interactions with other proteins may have a greater influence on its turnover rate than MatP does.

**Table 2:** Half-time for fluorescence recovery of FtsZ, ZapA and ZapB.

	n	BW25113	Δ*matP*
FtsZ	58	12.3 ± 0.2s	10.5 ± 0.3s
ZapA	51	14.2 ± 1.1s	5.7 ± 0.2s
ZapB	59	19.8 ± 1.0s	11.6 ± 0.4s

*Errors are from se of bootstrapping.

We note that these FRAP measurements were obtained under slow growth conditions, where the loss of MatP has little to no observable effect on the localization of FtsZ [14]. Nevertheless, here we observed significant effects on the turnover dynamics for ZapA and ZapB structures. These results suggest that MatP modulates the dynamic turnover of all three protein structures even in the absence of obvious mislocalization phenotypes.

### Δ*matP* cells have shorter constriction periods

The FtsZ-ring has long been regarded as not only the critical structural component of the divisome, but also a primary driving force for cell constriction [6,45]. If structural stability of the FtsZ-ring is important for active force generation, constriction may be slowed in cells where the structural stability of the FtsZ-ring is compromised by disruption of the FtsZ-ZapA-ZapB-MatP network. To test this idea we measured the constriction period, *τ*_c_, in wt and Δ*matP* cells ectopically expressing similar low levels of FtsZ-GFP under our slow growth condition (S10 Fig) using time-lapse fluorescence microscopy (Methods). We defined constriction initiation as the time when an indentation of cell wall was first visible in bright-field images, and the end of constriction as the time when FtsZ-GFP fluorescence completely disappeared from, and did not return to, the midcell (S11 Fig). Surprisingly, we found that the constriction periods of Δ*matP* (40.3 ± 2.6 min, n = 37;$\;\overset{-}{x}$ ± se) and Δ*matP*Δ*slmA* cells (34.3 ± 2.2 min, n = 50) were significantly shorter than that of the wt cells (48.8 ± 3.0 min, n = 44, p < 0.04), while the doubling time remained similar (~200 min, Table 3). These results are contrary to our expectation that FtsZ-ring stability promotes constriction progress. As we will discuss below, two possible mechanisms coordinating the rate of constriction and nucleoid segregation could contribute to this observed phenomenon.

**Table 3:** Time-lapse analysis of FtsZ-GFP in mutant strains.

			constriction	constriction period
	n	cell cycle (min)	period (min)	(% of cell cycle)
BW25113	44	199.2 ± 11.5	48.8 ± 3.0	27 ± 2
Δ*matP*	37	208.2 ± 9.2	40.3 ± 2.6	20 ± 1
Δ*matP*, Δ*slmA*	50	196.9 ± 12.6	34.3 ± 2.2	18 ± 1

## Discussion

### Structural and spatial arrangement of the FtsZ-ZapA-ZapB-MatP network

**Fig 6:**
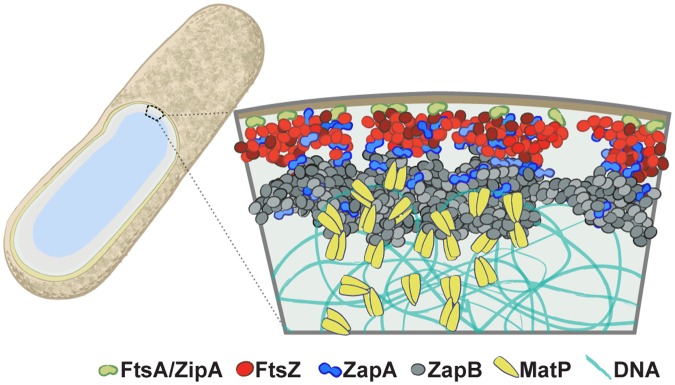
The multi-layered organization of the *E*. *coli* divisome. A schematic illustrating the relative radial arrangement of FtsA/ZipA-FtsZ-ZapA-ZapB-MatP. FtsA (green) and ZipA (orange) tether the punctuate FtsZ (red) structure to the inner membrane. ZapA (blue) mimics FtsZ but can deviate, possibly by interacting with ZapB or a number of membrane proteins [50,51]. The large, internal ZapB (grey) structure indirectly associates with FtsZ through ZapA, and is anchored on the chromosome through its associations with MatP (yellow).

In this work, we provided a quantitative characterization of the spatial organization and function of the FtsZ-ZapA-ZapB-MatP network in *E*. *coli* cells. By taking advantage of the superior spatial resolution and detection sensitivity offered by single-molecule based superresolution imaging, we quantified the spatial arrangement of each protein and showed that they are positioned to form a large, multi-layered network extending from the membrane to nucleoid at the midcell. We found that the FtsZ-ring comprises a heterogeneous arrangement of FtsZ clusters and is displaced on average 13 nm away from the cytoplasmic face of the inner membrane. ZapA adopts a similar heterogeneous clustered arrangement to that of FtsZ and resides at a similar radial plane. Interestingly, ~20% of ZapA structures deviated from FtsZ structures, and in a few cases ZapA clusters appeared to be sandwiched in between FtsZ clusters. ZapB forms wider, larger and more cohesive structures that are displaced on average ~40 nm internal to FtsZ and ZapA. Finally, MatP forms puncta with an average diameter of ~100 nm and is located on average ~30 nm internal to ZapB, or ~280 nm away from the center of the cell. As MatP binds to the *ter* region of the chromosome, it is reasonable to expect that its position also reflects that of the *ter* region of the nucleoid at midcell. From these measurements a three-dimensional architecture of a multi-layered protein network begins to emerge (Fig 6). As we will discuss below, these quantitative measurements provide an important physical framework upon which mechanisms regarding constrictive force generation, divisome assembly, FtsZ-ring function, and the interplay between cell wall constriction and chromosome segregation should be considered.

### Structural deviations of ZapA and ZapB from FtsZ

Historically, the FtsZ-ring has been regarded as the main structural component of the divisome, serving as a scaffold for the assembly of all other division proteins at the constriction site [46]. Hence, its structural organization was thought to determine that of the other divisome components through a complex interaction network. We and others previously showed that the FtsZ-ring is not a smooth, uniformly organized structure, but rather a heterogeneously arranged assembly of FtsZ clusters [10–15]. Here, we show that ZapA also displays a heterogeneous organization that morphologically mimics the FtsZ-ring. This heterogeneity was in contrast to the largely uniform appearance of ZapB-mEos2, indicating that the heterogeneous nature of FtsZ and ZapA is specific to their assembly.

We additionally show that the structures of both ZapA and ZapB can significantly deviate from those of FtsZ using two-color PALM. These observations suggest that, although the midcell localization of ZapA and ZapB is dependent on FtsZ, their structures do not replicate that of FtsZ, and there is unlikely a strictly-defined protein complex with fixed stoichiometry between FtsZ, ZapA and ZapB. Note that in a recent superresolution study, structural deviations of FtsA and ZipA from FtsZ were also observed [47], suggesting that this may be a common feature of divisome assembly. Although no defined complex appears to predominate, we find it interesting that the radial separation between FtsZ and ZapB is conserved throughout constriction (Fig 2E). We reason that maintaining the relative spatial arrangement between FtsZ and ZapB structures may aid the efficiency of constriction.

What leads to the apparent structural deviations of ZapA and ZapB from FtsZ? It is likely that the inherent oligomerization properties of these proteins coupled with their interactions with other division proteins play a large role. *In vitro* biochemical studies have shown that FtsZ polymers exhibit remarkable polymorphism, assembling into single-stranded protofilaments, sheets, bundles, helices and toroids [48]. ZapA itself does not extensively polymerize but exists in a dimer-tetramer equilibrium [49]; ZapB, on the contrary, readily forms large, bundled polymers [19]. This self-polymerization capability of ZapB likely contributes to its ability to substantially deviate from FtsZ structures.

The protein interactions exhibited by FtsZ, ZapA, and ZapB are also consistent with their locations relative to FtsZ. Bacterial two-hybrid and *in vivo* FRET have shown that FtsZ mainly interacts with FtsA, ZipA, ZapA, and FtsK, while ZapA associates with a large number of inner membrane proteins that do not interact with FtsZ, including: FtsQ, FtsL, FtsB, FtsW and FtsN [50–52]. Notably, these latter proteins are involved in septum synthesis during cell wall constriction. The membrane-proximal location of ZapA is conducive to interactions between ZapA and these proteins, and these interactions may facilitate the constriction progress. It will be interesting to apply the tools developed in this study to investigate the degree of colocalization of other divisome proteins with FtsZ and ZapA. Different colocalization patterns may reflect specific roles for FtsZ and ZapA in supporting these proteins’ functions in cell wall constriction, further elucidating the relationship between the structural organization and function of the divisome.

ZapB does not exhibit the interaction promiscuity of ZapA, and has only been shown to interact with ZapA and MatP [26,29]. These limited protein-protein interactions are consistent with the fact that, compared to ZapA, ZapB is displaced an additional ~40 nm into the cytoplasm, near MatP. It is likely that the morphology and localization of FtsZ, ZapA and ZapB structures are greatly influenced by their respective interacting partners, and that these interactions are not uniformly distributed across the structures.

### Role of the FtsZ-ZapA-ZapB-MatP network in positioning the division plane

Correctly positioning the FtsZ-ring at the midcell in *E*. *coli* is largely attributed to two negative regulatory systems: MinCDE and SlmA [53]. MinCDE is a three-component system that oscillates from pole to pole, preventing polar FtsZ-ring formation [54]. SlmA is a DNA-activated FtsZ antagonist that prevents FtsZ-ring formation over the bulk nucleoid regions except the *ter* macrodomain [55]. Together, these two systems create a midcell zone where the FtsZ-ring can stably polymerize. Here we show that a third system, the ZapA-ZapB-MatP network, also contributes to the midcell positioning of the FtsZ-ring by providing a physical tether to the *ter* region of the chromosome. Disabling this linkage by deleting any one of the three proteins leads to dispersed FtsZ clusters in a large region around the midcell [14]. Importantly, the effect in the absence of MatP is not solely mediated by abnormally distributed SlmA, as FtsZ mislocalization persists in a *ΔmatPΔslmA* strain. We postulate that as the *ter* region moves toward the midcell at the beginning of cell cycle and resides there until the end of DNA replication [28], it provides a convenient positive positioning system to reinforce the midcell localization of the divisome—MatP promotes the localization of ZapB to midcell through its direct interaction with ZapB, which in turn influences the localization of ZapA and further FtsZ. This mechanism is supported by recent work from the Männik and Sherratt groups, who showed that the FtsZ-ring colocalizes with the MatP-bound *ter* macrodomain at the center of nucleoid in cells depleted of both MinC and SlmA [30]. Interestingly, this colocalization was diminished, but not completely abolished, in the absence of all the three systems, suggesting the presence of other positioning systems. The variety and redundancy of positioning systems highlights the importance of the FtsZ-ring and the robust nature of its highly-evolved regulatory system.

### Role of the FtsZ-ZapA-ZapB-MatP linkage in coordinating division with nucleoid segregation

One unexpected finding of this study is that the deletion of *matP* leads to a faster cell wall constriction rate. This finding is counter-intuitive, because we have shown that the presence of MatP and the associated protein network helps to position and maintain the FtsZ-ring at the midcell, and the deletion of MatP leads to mislocalized FtsZ-ring and faster turnover dynamics of ZapA and ZapB. If the stability of the FtsZ-ring is indeed essential for efficient cell wall constriction, we should expect the deletion of MatP to slow down constriction, instead of speeding it up.

One possible explanation for this apparent paradox is that the cell wall constriction machinery has the ability to proceed much faster than nucleoid segregation. However, under normal conditions, cell wall constriction does not proceed at its maximum speed because some divisome constituents may exist to impede, or slow down cell wall constriction to allow time for nucleoid segregation. If the rates of the two processes are not balanced, a septum could form over an unsegregated nucleoid, which is detrimental for cell division.

How would MatP exert its influence on cell wall constriction rate? This influence could be transmitted through the network of FtsZ-ZapA-ZapB-MatP, or by the altered distribution of SlmA on the nucleoid. We showed in this study that the effects of Δ*matP* on FtsZ mislocalization and constriction rate remained in the *ΔmatPΔslmA* double deletion strain, suggesting that the physical linkage of FtsZ-ZapA-ZapB-MatP indeed plays a direct role. We propose that there could be two different mechanisms mediated by the linkage to explain the influence of MatP on cell wall constriction rate.

In the first mechanism, the physical linkage between the membrane and nucleoid by the FtsA-FtsZ-ZapA-ZapB-MatP protein network could act as a steric hindrance to prevent cell wall constriction from proceeding too fast. As both ends of the linkage are anchored, this protein network effectively couples cell wall and nucleoid segregation mechanically so that cell wall constriction can only complete when the *ter* macrodomains are resolved at the end of DNA replication and moved outside of the midcell. If this linkage is disabled, unchecked cell wall constriction may not always allow sufficient time for complete nucleoid segregation, perhaps explaining the occasional nucleoid segregation defects observed in cells lacking MatP [28]. This mechanism is similar to one previously proposed for FtsK, which can interact with divisome proteins (FtsZ, FtsL, FtsQ) and the chromosome [56]. A recent study found that the ordered segregation of sister chromosomes by FtsK requires the presence of MatP, suggesting that these two proteins may coordinate with each other [57].

An alternative mechanism could be that the additional stability provided by the presence of the ZapA-ZapB-MatP network actually inhibits the ability of FtsZ to exert an active force to drive cell wall constriction. The inhibitory effect of an overly-stable FtsZ-ring is supported by the lethality of FtsZ overexpression [58]. In this mechanism, MatP facilitates the midcell localization of ZapA by interacting with ZapB, which further promotes the inhibitory bundling effect of ZapA on FtsZ. *In vitro* it has been shown that ZapA promotes FtsZ polymerization [18,24]. We and others showed that ZapA and ZapB promote FtsZ-ring assembly *in vivo* by aligning and corralling FtsZ polymers at the midcell. It may be possible that a less bundled, highly dynamic FtsZ-ring could be more active in directing/driving cell constriction.

One possible way to differentiate the above two mechanisms would be to examine the constriction rate in a Δ*zapA* or Δ*zapB* strain. If the physical, steric hindrance mechanism has a larger role, disabling the linkage by deleting *zapA* or *zapB* should have the same effect as deleting *matP*. If the activity inhibition mechanism has a larger role, deleting *zapA* or *zapB* should have a larger impact on cell wall constriction rate. This is because ZapA and ZapB can still localize to the midcell to inhibit FtsZ activity in the absence of MatP, albeit less efficiently. Previously we have observed that a subpopulation of Δ*zapA* and Δ*zapB* cells had much faster cell cycle time than wt cells, which we attributed to the ability of rapid reinitiation of previously primed division sites. We were unable to quantitatively verify whether the constriction rates of these cells were indeed faster than that in Δ*matP* cells due to the highly dynamic nature of FtsZ-ring and abnormal septum formation in Δ*zapA* and Δ*zapB* cells. It would be interesting to design further experiments to examine these hypotheses.

## Materials and Methods

### Bacterial strains, growth conditions and materials

Bacterial strains and plasmids are indicated in S2 Table. Construction of strains and plasmids is detailed in the S1 Text and primers used are listed in S3 Table. Prior to imaging, all cells were grown from a single colony in LB media overnight at 37°C. For our default slow growth condition, cells were then diluted in M9 minimal media supplemented with 0.4% Glucose, MEM Vitamins and MEM Amino Acids (M9^+^), and grown at room temperature (RT) for at least 20 hrs. For our fast growth condition, cells were diluted in EZ Rich Defined Media (Teknova) supplemented with 0.4% Glucose and incubated at 37°C. When appropriate, we added 150 μg ml^-1^ chloramphenicol, 50 μg ml^-1^ kanamycin, 50 μg ml^-1^ carbenicillin or 100 μg ml^-1^ spectinomycin. Expression of FtsZ-mEos2 (pJB042) and mEos2-ZapA (pJB051) was induced with 20 μM IPTG for 2 hrs. Expression of ZapB-mEos2 (pJB045) and MatP-mEos2 (pJB128) was induced with 5–10 or 33 μM IPTG for 1hr, respectively. The dual-labeled Dronpa-ZapA—FtsZ-PAmCherry1 (pJB089) and Dronpa-ZapA—PAmCherry1-ZapA (pJB090) strains were induced with 20 μM IPTG for 2 hrs. For the ZapB-FtsZ 2C-PALM sample, FtsZ-PAmCherry1 (pJB066) was induced with 0.2% Arabinose for 30 min, while ZapB-Dronpa (pJB073) was uninduced. For all constructs, induction was followed by a washing step and a 2–3 hr outgrowth at RT without inducer. Fixation for iPALM samples was performed using 4% (v/v) formaldehyde in PBS (pH 7.4) for 45 min at RT.

### Fluorescence imaging

All ensemble and PALM image acquisition and sample preparation were performed as described previously [10,59]. All PALM images were constructed from 3,000 frames acquired at a frame rate of ~100 s^-1^ with a constant 405 nm activation (~5 W cm^-2^). Single-molecule identification and image reconstruction were described previously [59]. Images are displayed in pseudo-color ('Red Hot') via ImageJ with a pixel size of 15 nm. Measurements of cell length and band width were performed using custom MATLAB (The MathWorks, Inc., Natick, MA) software and are described elsewhere [10,59]. Diameters of band-like structures were determined by first projecting the band intensity along the short axis of the cell. Peak intensities were then identified using the 'findpeaks' MATLAB function and the distance between the two most distal peaks was calculated. Immuno-based superresolution (STORM)[60] imaging of FtsZ was performed with Alexa Fluor 568 Goat Anti-Rabbit IgG (Invitrogen, GAR-568), as described previously with α-FtsZ (a gift from H. Erickson). STORM imaging of ZapB was performed similarly with α-ZapB (a gift from K. Gerdes) and GAR-568 applied at 1:500 and 1:1,000, respectively.

Two-color PALM imaging utilized the OptoSplit II (Andor) device, which simultaneously projected the two emission signals onto separate halves of the CCD chip. We obtained 1,500 frames at a rate of 100 s^-1^ using 405-nm activation at ~500 mW cm^-2^ followed by a second 1,500-frame acquisition at ~5 W cm^-2^. Both acquisitions employed a constant 488-nm and 561-nm excitation at a power density of ~1 kW cm^-2^.

Image overlay of the two-color images employed a transformation step that was achieved by using the multi-colored emission spectrum of 100 nm TetraSpeck beads (Life Technologies, Inc.), as described previously [61]. Briefly, we acquired hundreds of snapshots of single TetraSpeck beads simultaneously in both channels at various positions across the imaging region, generating a large dataset of control points. We then calculated the transformation of two-color data using these control points and the 'cp2tform' function in MATLAB [62]. This type of global transformations resulted in ~18-nm registration error in our microscope setup.

iPALM imaging and sample preparation was performed as described previously [37,38] with the following exceptions. Gold-embedded coverslips were coated with 0.1% Poly-L-Lysine (Ted Pella) for 40 min, washed with PBS (pH 7.4), and dried with purified air. Alexa Fluor 568 carboxylic acid (Life Technologies, Inc.) was diluted (~2e^-9^) in PBS (pH 7.4), applied to the coverslip for 15 min, and then washed and dried as above. Fixed cellular samples were subsequently applied in a similar fashion. Each image was produced from 45,000-100,000 frames. The average spatial resolution obtained for the x-, y- and z-axes was 21 nm, 23 nm, and 17 nm, respectively.

We obtained at least three biological replicates for each fusion protein on different days. iPALM data were processed via the PeakSelector v9.3 software [37] to extract molecular coordinates and fitting errors. Further analysis was performed by custom MATLAB software. Specifically, for each iPALM image, we first determined the z-position of the coated coverslip surface by fitting the Alexa Fluor 568 signal to a Gaussian function. This fit was typically characterized by a FWHM of ~10 nm. We then drew a user-defined box centered at the bottom of the cell, which in general had a z-depth of 150 nm and width of 200 nm to avoid molecules along the curved regions (Fig 3). The z-positions of all molecules in this box were then averaged and the relative distance to the coverslip was taken as the mean z-position of the protein in the cell.

### Estimating mEos2 fusion concentration

We used the fluorescence intensity of mEos2 after excitation with a 488-nm laser to quantify the relative expression levels of FtsZ-mEos2, mEos2-ZapA and ZapB-mEos2. We used custom MATLAB software to measure the integrated cellular intensity (A.U.) of each fusion protein: FtsZ = 46,000 ± 2,000, ZapA = 94,000 ± 5,000, and ZapB = 40,000 ± 3,000 ($\overset{-}{x}$ ± se). Taking into account the previously determined expression level of FtsZ-mEos2 under the same growth conditions (30% of FtsZ_total_) [14] and the previously determined endogenous concentrations of the three proteins (FtsZ ≈ 5,000, ZapA ≈ 5,000 and ZapB ≈ 15,000 molecules cell^-1^) [10,14,18,19,63], we estimate that the fusions are present at 30%, 45% and 10% of the total (wt + fusion) protein concentration.

### Cross-correlation analysis of 2C-PALM images

We used a previously published coordinate-based cross-correlation analysis to identify the apparent displacement between different protein pairs in 2C-PALM images [34,35]. Briefly, regions around the midcell of each 2C-PALM image were cropped and the cross-correlation between the two proteins of interest was calculated as a function of displacement along the short axis of the cell. Apparent displacement between a given protein pair in a single cell was defined as the displacement value that exhibited maximal cross-correlation. Fig 2D shows the distributions of this value for FtsZ-ZapA, FtsZ-ZapB and ZapA-ZapA pairs. The mean apparent displacement value for ZapA-ZapA (33 nm) reflects our resolution in determining the displacement between two protein structures.

### Fluorescence recovery after photobleaching

Fluorescence recovery after photobleaching (FRAP) was carried out on wild-type BW25113 cells or Δ*matP* mutants (JW0939) ectopically expressing FtsZ-GFP (pXY027), GFP-ZapA (pJB154) or GFP-ZapB (pJB150) in the absence of inducer. Imaging was conducted on cells immobilized on 3% Agarose gel pad (M9^+^ without MEM Vitamins) using an Olympus IX-71 inverted microscope with excitation from a 488-nm laser (OBIS, Coherent, Santa Clara, CA). The excitation laser was passed through a linear polarizer (Thorlabs, Newton, NJ), expanded to a 10mm diameter, and split with a polarizing beamsplitting cube (Thorlabs, Newton, NJ) to generate two excitation beams. The reflected beam was then focused with an achromatic doublet lens (f = 750.0 mm, Ø2", Thorlabs, Newton, NJ) to allow epi-fluorescence illumination and recombined with the transmitted beam by another polarizing beamsplitting cube. Both beams illuminated the sample through a 100×, 1.45 NA TIRFM objective. The transmitted beam was focused by the objective to a ~300 nm radius spot for photobleaching and controlled by an independent shutter. The epi-fluorescence imaging beam (reflected beam) was focused to a radius of ~50 μm. The intensity of the photobleaching beam and imaging beam were 2 kW/cm^2^ and 5 W/cm^2^, respectively.

For imaging, the focus of the photobleaching beam was positioned to the midcell periphery of an individual cell. Timelapse fluorescence images were then acquired with the imaging beam at a rate of 30 min^-1^ for 4 min. The photobleaching step occurred during the second timelapse acquisition, all other frames were acquired using epi-fluorescence illumination. The exposure time for all acquisitions was 50 ms.

For FRAP analysis, we cropped the bleached area and whole midcell region and plotted their average intensity in each frame against time: *I*_*Bleach*_(*N*),*I*_*Midcell*_(*N*), where N is the frame number. We calculated the bleaching ratio as (*I*_*Bleach*_ *(1)-I*_*Bleach*_ *(3))*/*I*_*Bleach*_ *(3))I*_*Bleach*_ *(1)*, and only used trajectories where this value was greater than 40%. We normalized individual trajectories to [0,1], with the first acquisition post-photobleach (*I*_*Bleach*_ (3)) set as 0. We typically observed large fluctuations at the tail end of the bleached area (*I*_*Bleach*_(*N*)) trajectories, whereas trajectories of the whole midcell region (*I*_*Midcell*_(*N*)) were much more stable. Consequently, we used the average intensity of last 60 frames of the whole midcell region (< *I*_*Midcell*_(*61*:*120*) >) as the maximum to normalize each bleach trajectory (*I*_*Bleach*,*Nor*_ *(N))*.

We averaged the normalized trajectories of each dataset across all cells from at least two days of imaging and estimated their recovery rates by fitting the data to single exponential functions. These normalized trajectories were then resampled 3000 times by bootstrapping. The resampled trajectories were then averaged and fit to obtain the recovery half-time. We then calculated the standard error from the distribution of bootstrapped recovery half-time.

### Time-lapse fluorescence imaging and analysis for constriction periods

Imaging of FtsZ-GFP in BW25113, Δ*matP* and Δ*matP*Δ*slmA* was performed using plasmid JW0093 [64]. Each strain was first inoculated from a fresh colony into LB, grown overnight at 37°C to saturation, then diluted 1:100 into M9^+^ media and grown overnight at 30°C to saturation. This saturated culture was then diluted 1:200 into fresh M9^+^ media and grown at RT until mid-log phase (0.2–0.4 OD_600_). This 3-step culture preparation ensured similar expression levels of FtsZ-GFP among all three strains (see S10 Fig). The midlog culture was deposited onto a 3% agarose gel pad made with M9^+^ lacking MEM vitamins. Bright-field and green fluorescence images were acquired at 5-minute intervals. Constriction onset was determined from bright-field images as the first frame with visible cell wall indentation (S11 Fig). To limit interference from adjacent cells, only cells located at the outer edge of the growing microcolony were analyzed. The end of constriction, and thus the end of the mother cell cycle, and beginning of daughter cell cycles, was determined by the complete and unrecovered loss of GFP fluorescence from midcell (S11 Fig). Measurement bias in identification of constriction onset and completion may lead to underestimation of constriction times because detection of cell wall indentation is diffraction-limited [65] and detection of fluorescence loss at constriction completion is limited by signal sensitivity. However, comparison of relative constriction times between the three strains remains valid as the same criteria were applied consistently between all three strains.

## Supporting Information

S1 Text Supporting text. Additional information is provided.
(DOCX)

S1 Fig Functional characterization of mEos2-ZapA and ZapB-mEos2. Overlaid bright-field and fluorescence images illustrate the characteristic midcell localization of mEos2-ZapA (pJB051, A-B) or ZapB-mEos2 (pJB045, D-E) in wt (A,D) or respective deletion strains (B,E). Scale Bars, 500 nm. (C,F) Cell length distributions for wt and mutant strains in the absence and presence of mEos2-ZapA (C) or ZapB-mEos2 (F) are presented as box plots where the mean is boxed by the 95% confidence interval and bounded by the standard deviation.
(TIF)

S2 Fig Non-band structures of ZapA, ZapB, and FtsZ. PALM images of mEos2-ZapA (top row), ZapB-mEos2 (middle) and FtsZ-mEos2 (bottom) illustrate the alternate morphologies common to all three protein species – peripheral focus (left), peripheral foci pair (middle), and multiple non-planar structures (right). All images are displayed in order of bright-field (i), ensemble fluorescence (ii), and PALM image (iii). Scale Bars, 500 nm.
(TIF)

S3 Fig Cell-length dependence of FtsZ, ZapA and ZapB morphology. The PALM images for each protein species are categorized according to the four observed structural morphologies and the cell length distribution for each category is plotted as the mean boxed by the 95% confidence interval bounded by the standard deviation. Overlap of two boxed regions indicates the two are not significantly different (p > 0.05).
(TIF)

S4 Fig Superresolution imaging of native ZapB and FtsZ via immunofluorescence. Wt cells labeled with α-ZapB (A-D) or α-FtsZ (E-H) and stained with an Alexa Fluor 647-conjugated secondary antibody (Life Technologies, Inc.) were imaged as described previously [33]. Images are displayed in the order of bright-field (i), ensemble fluorescence (ii) and superresolution image displayed in pseudocolor (iii). Approximate cell outlines are indicated by yellow dashed lines. Scale Bars, 500 nm.
(TIF)

S5 Fig Functional characterization of two-color PALM fusion proteins. Representative snapshots for wt (A-C,E,G) or deletion strains (D,F,H) expressing FtsZ-PAmCherry1 (B), Dronpa-ZapA (C-D), ZapB-Dronpa (E-F) or PAmCherry1-ZapA (G-H) under slow growth conditions. All images are displayed as ensemble fluorescence images overlaid with bright-field images. (I) Box plots representing cell length distributions are presented as the mean length boxed by the 95% confidence interval bounded by the standard deviation. Scale Bar, 2 μm.
(TIF)

S6 Fig Influence of MatP on FtsZ midcell localization. Top: Bright-field and ensemble fluorescence images of wt, Δ*matP*, Δ*slmA*, and Δ*matP*Δ*slmA* cells expressing FtsZ-GFP under fast growth conditions. Similar FtsZ mislocalizations were observed both in Δ*matP* and Δ*matP*Δ*slmA* strains. Bottom: Table displaying cell length and percent of cells displaying abnormal FtsZ localization for all four strains.
(TIF)

S7 FigAnalysis of MatP cluster size.A threshold-based cluster analysis [14] was applied to PALM images of wt or Δ*matP* cells expressing MatP-mEos2. We determined the major axis length for each cluster and plotted the distribution as a histogram (blue bars). We found that the average major axis length of MatP-mEos2 clusters was 100 ± 58 nm ($\overset{-}{x}$ ± sd). Given that the average eccentricity of MatP-mEos2 clusters was 0.8 (near circular), this measurement represents an estimation of MatP-mEos2 cluster diameter.(TIF)

S8 Fig Displacement of MatP clusters along cellular axes. (A) Schematic of measurement geometry. We measured the distance of each MatP-mEos2 cluster (red circle) from the middle of the cell along the short (*r’*) and long (*l*) cellular axes (dashed lines). We binned these distances according to cell length and found that MatP clusters undergo a cell-length-dependent migration towards the middle of the cell along the long axis (B). We also found that MatP-mEos2 clusters remain tightly distributed along the short axis with an average displacement of 180 nm (C). Since PALM images are two-dimensional projections of three-dimensional objects (A), the average measured displacement (r') is related to the true radial displacement (r) as described in the S1 Text.
(TIF)

S9 Fig Modeling MatP radial displacement distributions. The distribution of distances (*r*’) separating MatP-mEos2 clusters from the middle of the cell along the short axis is plotted in 60 nm bins (yellow bars, n = 613; see S1 Text). (A) Short axis displacement distributions from three simulations using a Gaussian-distributed radius (r ± sd) and a randomly distributed projection angle (θ) are shown as dotted lines (n = 1000). These simulations were generated with the following parameters: *r* = 150 ± 100 nm (magenta), 250 ± 100 nm (red), or 350 ± 100 nm (cyan). We found that the radial distribution of MatP-mEos2 clusters was best fit with a true radius of 280 ± 120 nm. (B) The experimental data (yellow) was also well fit by an alternate model (orange) that assumed MatP could uniformly sample the cross-section of a nucleoid with a maximum radius of *r*_*max*_. Least squared fitting found the best-fit *r*_*max*_ to be 419 nm, resulting in a distribution defined by 279 ± 97 nm (*r* ± sd).
(TIF)

S10 Fig Expression level of FtsZ-GFP. A representative immunoblot of BW25113, Δ*matP*, Δ*matP*Δ*slmA* cells expressing FtsZ-GFP (pJW0093) stained with α-FtsZ. Signals from FtsZ-GFP and FtsZ_wt_ were quantified and used to calculate the fraction of FtsZ-GFP relative to that of FtsZ_wt_. A table summarizing the quantifications is displayed at the bottom.
(TIF)

S11 Fig Time-lapse imaging of FtsZ-GFP to measure constriction periods. Fluorescence and bright-field images of wt BW25113 (A) and Δ*matP* (B) cells expressing FtsZ-GFP (pJW003). Images are displayed as a time-lapse montage with the time-stamp of each image pair indicated at the top right in minutes. White arrows indicate loss of fluorescence from midcell (i.e. beginning/end of cell cycle). Red arrows indicate initiation of cell wall constriction.
(TIF)

S12 Fig Schematic for FtsZ linker length estimation. (A) The configuration of the FtsZ linker in the stretched (orange lines) or relaxed (orange coils) state when the C-terminal side (light shade) of FtsZ globular domain (yellow boxes) faces the membrane (see S1 Text). (B) The FtsZ linker configurations when the C-terminal side of FtsZ globular domain faces cytosol according to a recent model [66]. mEos2 (red) and membrane attached FtsA (blue) are drawn to scale. Corresponding distance estimates are labeled individually.
(TIF)

S1 Table PALM morphology frequency. All PALM images for FtsZ-mEos2, mEos2-ZapA, and ZapB-mEos2 were grouped according to their qualitative appearance. Representative images for non-band structures (i.e. focus, foci and helix) are displayed in S2 Fig, while band structures of non-constricting cells only are shown in Fig 1.
(DOCX)

S2 Table Strains and plasmids. A list of strains and plasmids used in this study.
(DOCX)

S3 Table Primer list. A list of primers used in this study.
(DOCX)
