# Supplementary material for: A Multi-layered Protein Network Stabilizes the Escherichia coli FtsZ-ring and Modulates Constriction Dynamics
Source: PLoS Genet. 2015 Apr 7;11(4):e1005128. doi: 10.1371/journal.pgen.1005128 (PMC4388696; doi:10.1371/journal.pgen.1005128)
Supplement: S1 Text — Additional information is provided. (DOCX) [file pgen.1005128.s001.docx]

Supplementary Text 1 for

**A multi-layered protein network stabilizes the *Escherichia coli* FtsZ-ring and modulates constriction dynamics**

Jackson Buss^1,3^, Carla Coltharp^1^, Gleb Shtengel^2^, Xinxing Yang^1^, Harald Hess^2^ and Jie Xiao^1,4^

^1^Department of Biophysics and Biophysical Chemistry, Johns Hopkins University School of Medicine, Baltimore, MD, USA; ^2^Janelia Farm Research Campus, Howard Hughes Medical Institute, Ashburn, VA, USA; ^3^Current Address: Department of Microbiology and Immunobiology, Harvard Medical School, Boston, MA, USA

^4^correspondence to: [xiao@jhmi.edu](mailto:xxxxx@xxxx.xxx)

**This PDF file includes:**

Supplementary Text 1

Supplementary Figs. 1-12

Supplementary Tables 1-3

Supplementary References

**Supplementary Text 1**

**Estimating the radial position of MatP**

We constructed a MatP-mEos2 fusion that appeared as distinct puncta when ectopically expression in a wild-type (wt) strain (Fig. 4A). These localizations were consistent with previous reports [1,2], and were similarly observed in a Δ*matP* strain, suggesting that our construct was a reliable label for wt MatP.

Characterization of MatP-mEos2 via iPALM was not possible. This is because iPALM used fixed cells, and the small population of DNA-bound MatP-mEos2 molecules became obscured by the larger population of DNA-unbound MatP-mEos2 upon fixation. In contrast, these fast-moving cytoplasmic species were undetectable by live-cell PALM. Therefore, we performed live-cell PALM imaging on cells ectopically expressing MatP-mEos2 to determine the radial position of MatP (Fig. 4A). We then applied a threshold-based clustering algorithm [3] to isolate individual clusters and measured the displacement of each cluster from midcell along the long axis (*ɭ*) and short axis (*r'*) of the cell (Supp. Fig. 8A).

We found that displacement of MatP-mEos2 clusters from the midcell along the long axis of the cell gradually decreased as cell length increased (Supp. Fig. 8B). This finding is consistent with previous results [1,2], which suggest that MatP moves with the *ter* region to midcell at late stages of DNA replication. In contrast, the short axis displacement (*r'*) remained largely constant at ~180 nm at different cell lengths, suggesting that the radial displacement of MatP did not change significantly during the cell cycle (Supp. Fig. 8C). Because the short axis displacement *r’* of a point is related to the true radial displacement (*r*) of the point by (Supp. Fig. 8A):

$r^{'}=r\cdot cos \theta$

*Eq. 1*

where *θ* is the angle between *r* and the imaging plane. The true *r* is related to the mean <*r*'> by:

$r =\frac{\sum_{i=1}^{n} r_{i}^{'}}{\sum_{i=1}^{n} \cos\theta_{i}}=\frac{\left\langle r' \right\rangle}{\left\langle\cos\theta\right\rangle}$

*Eq. 2*

When the angle *θ* is randomly and sufficiently sampled from 0 to 2π, $\left\langle\cos\theta\right\rangle$ approaches a fixed value at ~0.63. Therefore, the true radial displacement *r* can be calculated as:

$r =\frac{\left\langle r' \right\rangle}{0.63} = \frac{180}{0.63} = 285$

*Eq. 3*

We next compared the distribution of *r'* to those generated by two different models for the distribution of *r*. In the first model, we assumed that the true radial displacement *r* was Gaussian-distributed with a standard deviation of *s.* This *r* was then randomly sampled (n = 1000) with an angle between 0 and 2π and projected to the imaging plane to give rise to a measured *r'* (Supp. Fig. 9A). Using this model, we applied least-square fitting to minimize the difference between the model-predicted *r'* histogram with the experimentally measured *r'* histogram (Fig. 4B). This procedure allowed us to identify the best-fit *r* and *s* as 280 and 120 nm respectively. In the second model, we assumed MatP could sample the entire nucleoid cross-section uniformly and defined a single parameter for the nucleoid radius (*r_max_*). Least squared fitting to the experimental data resulted in *r_max_* of 419 nm (Supp. Fig. 9B). The <*r*> and *s* for this best fit model were 279 and 97 nm. The mean *r* values from both models are consistent with the mean calculation from *Eq. 3*, indicating that this calculation is robust to the underlying *r* distribution.

**Estimating the length of FtsZ’s C-terminal linker in relaxed state**

To estimate the relaxed length of the intrinsically disordered linker of *E. coli* FtsZ, Gardner *et al*. applied a worm-like chain model with a persistence length of 0.55 nm and estimated that the average end-to-end length of the linker in its relaxed state is 4.4 nm [4]. Using larger persistence lengths of 0.8 to 1.0 nm as measured for intrinsically disordered proteins in single-molecule pulling experiments [5,6] changes the end-to-end length to 5.2 ~ 5.8 nm. Therefore, we rounded the numbers and used 5 nm as the estimated length of the linker when in its relaxed state. When fully stretched, this linker would have a contour length of 17 nm (50 x 0.34 nm per amino acid).

**Deducing FtsZ z-position from that of its fused mEos2 label**

To estimate the distance between the polymerized globular domains of FtsZ protofilaments and the inner membrane from iPALM measurements, we must first estimate the distance between the globular domain of FtsZ and the mEos2 fluorescent label. The mEos2 label is attached the C-terminus of FtsZ, potentially blocking its accessibility to FtsA or ZipA. We previously showed that the FtsZ-mEos2 fusion protein can only localize to the midcell in the presence of unlabeled FtsZ [7], suggesting that the FtsZ-mEos2 fusion protein does not bind to the membrane tethers FtsA or ZipA by itself. Therefore, we reason that the disordered linker of the FtsZ-mEos2 fusion protein likely adopts the relaxed state, as it lacks a direct tether to the membrane. Thus, mEos2 is separated from C-terminal side of FtsZ's globular domain by the length of the relaxed linker (~5 nm). Note here that we do not treat the extreme C-terminal tail (~15 aa in *E. coli*) explicitly because this part of FtsZ is also unstructured and only becomes structured (~1 nm in length) upon binding to FtsA or ZipA

[8].

Using iPALM we measured that FtsZ-mEos2 is displaced on average 13 nm away from the cytoplasmic face of the inner membrane, which was approximated by the mEos2-MTS_Bs_ fusion protein. Assuming that the C-termini of FtsZ subunits face the membrane (Supp. Fig. 12A), the C-terminal side of FtsZ’s globular domain is then 22 nm away from the membrane (13 nm_IM<->mEos2_ + 4 nm_mEos2_ + 5 nm_linker_). Note that this 22 nm would be the maximal distance of the globular domain of FtsZ to IM according to the linear geometry we assumed between mEos2 and FtsZ. Taking into account the size of FtsA, we estimate that the linker would be stretched an additional 11 nm to a length of 16 nm (5 nm_linker_ + 11 nm = 16 nm; Supp. Fig. 12A). If the C-termini of FtsZ subunits face away from the membrane, then the linker would fold back onto the globular domain of FtsZ, as modeled in a recent study [9]. In this case the linker would be similarly stretched to 16 nm, as moving the N-terminus of the FtsZ globular domain closer to the membrane does not affect the linker position (Supp. Fig. 12B).

**How much force can FtsZ protofilaments generate?**

The FtsZ-ring is acknowledged as a force generator because membrane-attached FtsZ polymers can deform liposomes *in vitro* without any additional protein factors [6]. *In vivo,* the FtsZ-ring most likely generates some force, but the mechanism and magnitude of this force remain unclear. One prominent proposed force generation mechanism is based on a conformational change at the interfaces between FtsZ subunits, which can switch from a straight conformation in the GTP-bound state to a curved conformation in the GDP-bound state [43,44]. When an FtsZ protofilament with a mixed composition of GTP/GDP-bound subunits is more curved than the membrane to which it is tethered, it can exert a bending force onto that membrane [42].

If FtsZ protofilaments do exert a bending force onto the membrane, the intrinsically disordered linker (~50 amino acids) between its polymerizing globular domain and the C-terminal tail that binds the membrane tethers, FtsA and ZipA [45], would be stretched. This is because the linker is suggested to function as an elastic spring that can extend when a force is applied [45]. Thus, we can estimate the magnitude of this force by estimating how long the linker is stretched, which can be done by comparing our measured FtsZ z-position to that expected when the linker is relaxed.

Our iPALM results indicate that FtsZ-mEos2 is on average 13 nm away from the cytoplasmic face of the inner membrane. When the connection between the mEos2 label and FtsZ's globular domain is taken into account (S1 Text), this distance corresponds to a 22 nm separation between the membrane and the globular domains of FtsZ protofilaments (13 nm_IM<->mEos2_ + 4 nm_mEos2_ + 5 nm_linker_ = 22 nm_iPALM_) (Supp. Fig. 12). When the FtsZ subunit has a relaxed linker, and is tethered to FtsA, the expected separation between the membrane and FtsZ's globular domain is 11 nm (5 nm_linker_ + 6 nm_FtsA_ = 11 nm_relaxed_) (Supp. Fig. 12) [45]. Thus, according to our measurements, the linkers of FtsA-tethered FtsZ subunits are stretched by an additional 11 nm (22 nm_iPALM_ - 11 nm_relaxed_). For simplicity, we did not consider subunits tethered to ZipA, as FtsA is more highly conserved and shown to directly affect the force generation and polymerization properties of FtsZ [46,47]. In addition, ZipA is reported to have a large, unstructured domain between its N-terminal transmembrane and C-terminal FtsZ-binding domains, and thus may not bear any force to stretch the FtsZ linker [45,48].

A previous electron cryotomography study in *Caulobacter crescentus* showed that the distance between the inner membrane and FtsZ filaments is 16 nm [49]. It is interesting to note that the longer linker sequence of *C. crescentus* FtsZ (~180 amino acids, corresponding to a relaxed length of ~7 nm) does not result in a significantly longer separation between FtsZ and the membrane. Further investigations are needed to fully dissect the function of the linker and how it contributes to the overall placement of FtsZ protofilaments in different organisms.

If we assume that the linker of *E. coli* FtsZ is indeed stretched by an additional 11 nm *in vivo*, how much force does this represent? Using a simple worm-like chain model that has been used to describe the force extension curves of intrinsically disordered proteins [50], we estimate that a considerable pulling force (~18 pN) is applied at each tethered FtsZ subunit. This estimated force is consistent with a computational simulation which estimated that each transition of an FtsZ subunit interface from the straight to curved conformation could generate 20 - 30 pN of force [44,51]. Additionally, the 18 pN force is within the predicted range of constriction forces that could accomplish cell division (8 - 80 pN), which was estimated by simulating cell wall elasticity and growth during cell division [52]. Thus, our measurements are consistent with a model in which FtsZ protofilaments do generate a force that is sufficient to support cell division.

We note that these back-of-envelope distance calculations are based on simplified geometric constraints and neglect many important factors, including: the orientation of the linker with respect to mEos2 and to the globular domain of FtsZ, interactions between FtsZ and other divisome proteins, the thickness of the FtsZ-ring, and the stiffness of FtsZ protofilaments *in vivo.* In addition, for the heterogeneous FtsZ-ring to generate a uniform constrictive force across the septum, modifications to the bending mechanism, such as the iterative pinching mechanism [49], may be required. Other force generation mechanisms such as the condensation model have also been proposed [53], and further experiments are required to examine these alternatives.

Lastly, because our iPALM measurements were limited to non-constricting cells, our force estimates represent the steady-state condition prior to constriction in which the force generated by FtsZ protofilaments is balanced by opposing forces (e.g. attachments between the inner membrane and cell wall). As such, we cannot assess whether the amount of force generated by FtsZ protofilaments relative to these opposing forces remains constant or changes upon constriction initiation. Thus, the key question of whether the FtsZ-generated force is indeed the main driving force for constriction initiation or progress remains to be examined.

**Dynamic maintenance of the division plane**

Using FRAP we show that FtsZ, ZapA and ZapB constantly undergo dynamic turnover with their respective cytoplasmic pools. The highly dynamic nature of FtsZ and its associated proteins at midcell has been well documented in previous studies [16,17]. FRAP experiments have shown that FtsZ shares similar turnover rates with FtsA and ZapA in both *E. coli* and *B. subtilis* [16,63]. Additionally, a recent live-cell superresolution imaging study showed that the dynamic rearrangement of EzrA and PBP2 clusters in *Staphylococcus aureus* occurs on a similar timescale to that of FtsZ [13]. The dynamic turnover of the FtsZ-ring and its associated proteins enables cells to quickly abort division in order to resolve other issues when cells encounter unfavorable growth conditions. Accordingly, it has been regarded as a key entry point for FtsZ-ring regulators such as SulA, SlmA, MinC and ClpX, which modulate FtsZ-ring assembly by acting on either the cytoplasmic, unincorporated pools of FtsZ or on the incorporated protofilaments [61,64-66].

The observed dynamic behaviors of FtsZ, ZapA and ZapB also raise the question of how such a highly dynamic network provides the necessary physical reinforcement to positively position the FtsZ-ring at midcell. One unique aspect we observed in this work may provide an important clue to this question. We observed that while ZapB also undergoes a dynamic turnover, its rate is approximately two-fold slower than that of FtsZ and ZapA. We suggest that the slower dynamics of ZapB may provide a temporary landing pad for FtsZ assembly, serving as a short-term memory for established FtsZ-ring positions. Transmission of changes in the FtsZ-ring structure (e.g. complete disassembly) to ZapB and possibly other divisome components is delayed by differences in their dynamic time scales. Under conditions that are favorable for growth and division, FtsZ can quickly reassemble and continue to support division due to the continuous presence of the ZapB structure. However, under unfavorable conditions where FtsZ is actively disassembled and does not return quickly, ZapB and other divisome components eventually follow suit and dissociate.

We note that in the absence of MatP, although the turnover rates of both ZapA and ZapB were significantly increased, under our slow growth conditions no obvious FtsZ mislocalization was observed. It is most likely that the positioning and stabilization of the FtsZ-ring at the midcell is the result of the concerted effort of the MinC, SlmA, and ZapA-ZapB-MatP systems. The effect of the latter may only be significant under fast growth conditions where rapid establishment of the division site by the correct localization of FtsZ-ring is needed. It is also possible that other interaction networks may serve as additional short-term memories for FtsZ localization, and it will be interesting to apply these studies of protein dynamics to other divisome proteins.

**Plasmid & Strain Construction**

Plasmid purification and gel-extraction was performed using spin-columns from Fermentas. All genes were amplified with the indicated primers (Supp. Table 3) using PfuUltra II Fusion HS DNA Polymerase from Agilent. All restriction enzymes, as well as the ligase (Quick Ligation Kit) were obtained from NEB.

pJB051 (P_T5-lac_::mEos2-ZapA) was constructed by amplifying *mEos2* and *zapA* from pJB042 and JW2878 (ASKA+, ZapA), using primer pairs 1-2 and 3-4, respectively. The fragments were restricted with SpeI and EcoRI or EcoRI and NotI, gel-purified, and ligated with pJB042 digested similarly with SpeI and NotI. The linker sequence is GSAGSAAGSGEF.

pJB045 and pJB091: *zapB* and *mEos2* were amplified from JW3899 (ASKA+, ZapB) and pJB042 with primer pairs 5-6 and 7-8, respectively. Fragments were restricted with SpeI and EcoRI or EcoRI and NotI, and ligated into pJB042 restricted with SpeI and NotI. ZapB-mEos2^1^ from pJB045 contains the linker sequence GSAGSAAGSGEF. A similar construct (pJB091, P_T5-lac_::ZapB-mEos2^2^) with the linker sequence SIPGA was constructed by amplification with primer pairs 5-9 and 10-8, restriction with SpeI and SfoI or SfoI and NotI, and ligation into pJB042 restricted with SpeI and NotI. The ZapB-mEos2^2^ construct is analogous to the previously characterized ZapB-GFP [10] constructs and was found to be indistinguishable from ZapB-mEos2^1^ (data not shown).

pJB057: *Dronpa* [11] and *zapA* were amplified from pDG1-S1 (Amalgaam) and pJW2878 (ASKA+, ZapA), using primer pairs 11-12 and 13-4, respectively. The fragments were digested with SpeI and NheI or NheI and NotI, gel-purified, and ligated with a pJB042 digested with SpeI and NotI. The P_T5-lac_::Dronpa-ZapA contains the linker sequence GSAGSAAGSGEF.

pJB073: *Dronpa* was amplified from pDG1-S1 (Amalgaam) with primer pair 14-15, restricted with SfoI and NotI, and ligated into a similarly digested pJB091.

pJB058 and pJB066: *PAmCherry1* [12] was amplified with primer pair 16-17, restricted with NotI and inserted into a similarly digested JW0093 (ASKA+, FtsZ) creating pJB058 (P_T5-lac_::6xHis-FtsZ-PAmCherry1). *ftsz-PAmCherry1* was amplified from pJB058 with primer pair 18-19, digested with NheI and SalI, and ligated into a similarly digested pJB056, creating pJB066 (P_BAD_::FtsZ-PAmCherry1). The linker sequence is GLCGRM.

pJB089: *ftsz-PAmCherry1* and preceding ribosome binding site (RBS) was amplified from pJB066 using primer pair 20-21, digested with HindIII and ligated into a similarly digested pJB057 generating P_T5-lac_::Dronpa-ZapA—FtsZ-PAmCherry1. The two open reading frames are shifted relative to one another.

pJB061 and pJB090: *PAmCherry1* was amplified from pJB058 with primer pair 23-24, digested with NheI, and ligated into a similarly digested pJB056 creating P_BAD_::PAmCherry1-ZapA. *PAmCherry1-zapA* and the preceding RBS were amplified from pJB061 with primer pair 20-25, restricted with HindIII, and ligated into a similarly digested pJB089 creating P_T5-lac_::Dronpa-ZapA—PAmCherry1-ZapA. The two open reading frames are shifted relative to one another.

pXY029: *mEos2* was amplified with primer pair 1-26. The resulting fragment was amplified with primer pair 1-27, restricted with SpeI and NotI, and ligated into a similarly digested pJB042. The membrane targeting sequence of MinD from *Bacillus subtilis* (VLEEQNKGMMAKIKSFFGVRS) was included in primers 26 and 27, resulting in P_T5-lac_::mEos2-MTS.

pXY027: *ftsZ-GFP* was amplified from JW0093 (ASKA+, FtsZ) with primer pair 18-28, digested with SacII and NotI, and ligated into a similarly digested pJB042 creating P_T5-lac_::FtsZ-GFP with a GLCGR linker sequence.

pJB043 and pJB128: *ftsZ-mEos2* was amplified from pJB042 with primer pair 29-30, restricted with XbaI and SnaBI, and ligated into a similarly digested pDR122 (a gift from P. de Boer) generating pJB043. The *matP* sequence was amplified from K12 genomic DNA using primers 31 and 32, restricted with XbaI and BamHI, and ligated into a similarly digested pJB043 resulting P_T5-lac_::MatP-mEos2.

JB636 was constructed from JW0939 (Keio) using standard lambda red technology [13] with SlmA(ttk)-specific primers [14].

pJB154: *gfp* was amplified using primer pair 33-34, restricted with SpeI and NheI, and ligated into a similarly digested pJB057.

pJB150: *gfp* and *zapB* was amplified with primer pairs 33-34 and 35-28, restricted with SpeI-NheI or NheI-NotI, and ligated into pJB057 digested with SpeI-NotI.

**Supplementary References**

1. Mercier R, Petit M-A, Schbath S, Robin S, Karoui El M, et al. (2008) The MatP/matS site-specific system organizes the terminus region of the E. coli chromosome into a macrodomain. Cell 135: 475–485. doi:10.1016/j.cell.2008.08.031.

2. Espeli O, Borne R, Dupaigne P, Thiel A, Gigant E, et al. (2012) A MatP-divisome interaction coordinates chromosome segregation with cell division in E. coli. The EMBO Journal 31: 3198–3211. doi:10.1038/emboj.2012.128.

3. Buss JA, Coltharp C, Huang T, Pohlmeyer C, Wang S-C, et al. (2013) In vivo organization of the FtsZ-ring by ZapA and ZapB revealed by quantitative super-resolution microscopy. Mol Microbiol 89: 1099–1120. doi:10.1111/mmi.12331.

4. Gardner KAJA, Moore DA, Erickson HP (2013) The C-terminal linker of Escherichia coli FtsZ functions as an intrinsically disordered peptide. Mol Microbiol 89: 264–275. doi:10.1111/mmi.12279.

5. Kim J, Zhang C-Z, Zhang X, Springer TA (2010) A mechanically stabilized receptor-ligand flex-bond important in the vasculature. Nature 466: 992–995. doi:10.1038/nature09295.

6. Miyagi A, Tsunaka Y, Uchihashi T, Mayanagi K, Hirose S, et al. (2008) Visualization of intrinsically disordered regions of proteins by high-speed atomic force microscopy. ChemPhysChem 9: 1859–1866. doi:10.1002/cphc.200800210.

7. Fu G, Huang T, Buss JA, Coltharp C, Hensel Z, et al. (2010) In Vivo Structure of the E. coli FtsZ-ring Revealed by Photoactivated Localization Microscopy (PALM). PLoS ONE 5: e12680. doi:10.1371/journal.pone.0012680.

8. Szwedziak P, Wang Q, Freund SM, Löwe J (2012) FtsA forms actin-like protofilaments. The EMBO Journal: 1–12. doi:10.1038/emboj.2012.76.

9. Li Y, Hsin J, Zhao L, Cheng Y, Shang W, et al. (2013) FtsZ Protofilaments Use a Hinge-Opening Mechanism for Constrictive Force Generation. Science 341: 392–395. doi:10.1126/science.1239248.

10. Ebersbach G, Galli E, Møller-Jensen J, Löwe J, Gerdes K (2008) Novel coiled-coil cell division factor ZapB stimulates Z ring assembly and cell division. Mol Microbiol 68: 720–735. doi:10.1111/j.1365-2958.2008.06190.x.

11. Habuchi S, Ando R, Dedecker P, Verheijen W, Mizuno H, et al. (2005) Reversible single-molecule photoswitching in the GFP-like fluorescent protein Dronpa. Proceedings of the National Academy of Sciences 102: 9511–9516. doi:10.1073/pnas.0500489102.

12. Subach FV, Patterson GH, Renz M, Lippincott-Schwartz J, Verkhusha VV (2010) Bright Monomeric Photoactivatable Red Fluorescent Protein for Two-Color Super-Resolution sptPALM of Live Cells. J Am Chem Soc 132: 6481–6491. doi:10.1021/ja100906g.

13. Datsenko KA, Wanner BL (2000) One-step inactivation of chromosomal genes in Escherichia coli K-12 using PCR products. Proceedings of the National Academy of Sciences 97: 6640–6645. doi:10.1073/pnas.120163297.

14. Baba T, Ara T, Hasegawa M, Takai Y, Okumura Y, et al. (2006) Construction of Escherichia coli K-12 in-frame, single-gene knockout mutants: the Keio collection. Mol Syst Biol 2. doi:10.1038/msb4100050.
