# Supplementary figures and images for: A Multi-layered Protein Network Stabilizes the Escherichia coli FtsZ-ring and Modulates Constriction Dynamics
Source: PLoS Genet. 2015 Apr 7;11(4):e1005128. doi: 10.1371/journal.pgen.1005128 (PMC4388696; doi:10.1371/journal.pgen.1005128)

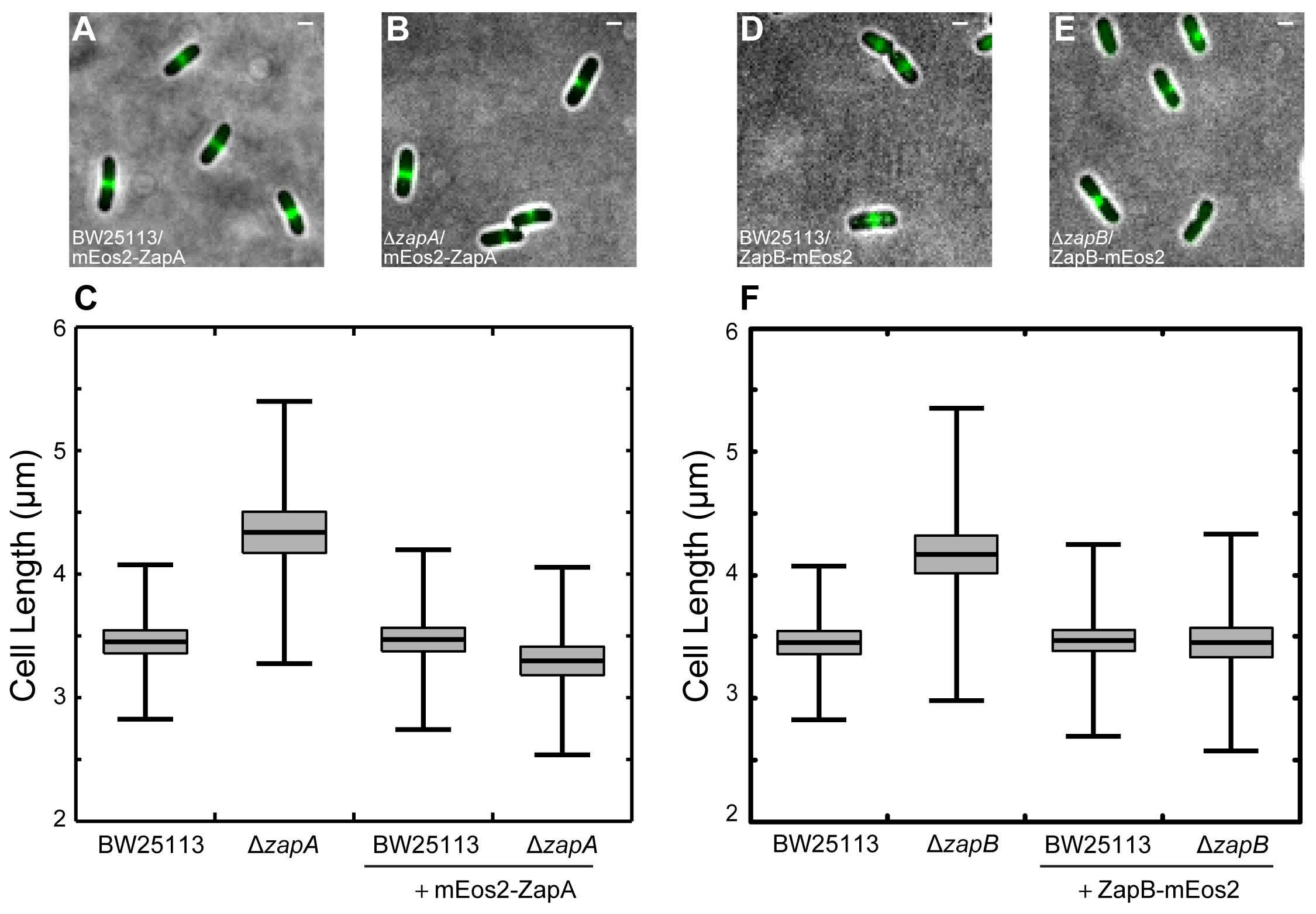

Supplement: S1 Fig — Overlaid bright-field and fluorescence images illustrate the characteristic midcell localization of mEos2-ZapA (pJB051, A-B) or ZapB-mEos2 (pJB045, D-E) in wt (A,D) or respective deletion strains (B,E). Scale Bars, 500 nm. (C,F) Cell length distributions for wt and mutant strains in the absence and presence of mEos2-ZapA (C) or ZapB-mEos2 (F) are presented as box plots where the mean is boxed by the 95% confidence interval and bounded by the standard deviation. (TIF) [file pgen.1005128.s002.tif]

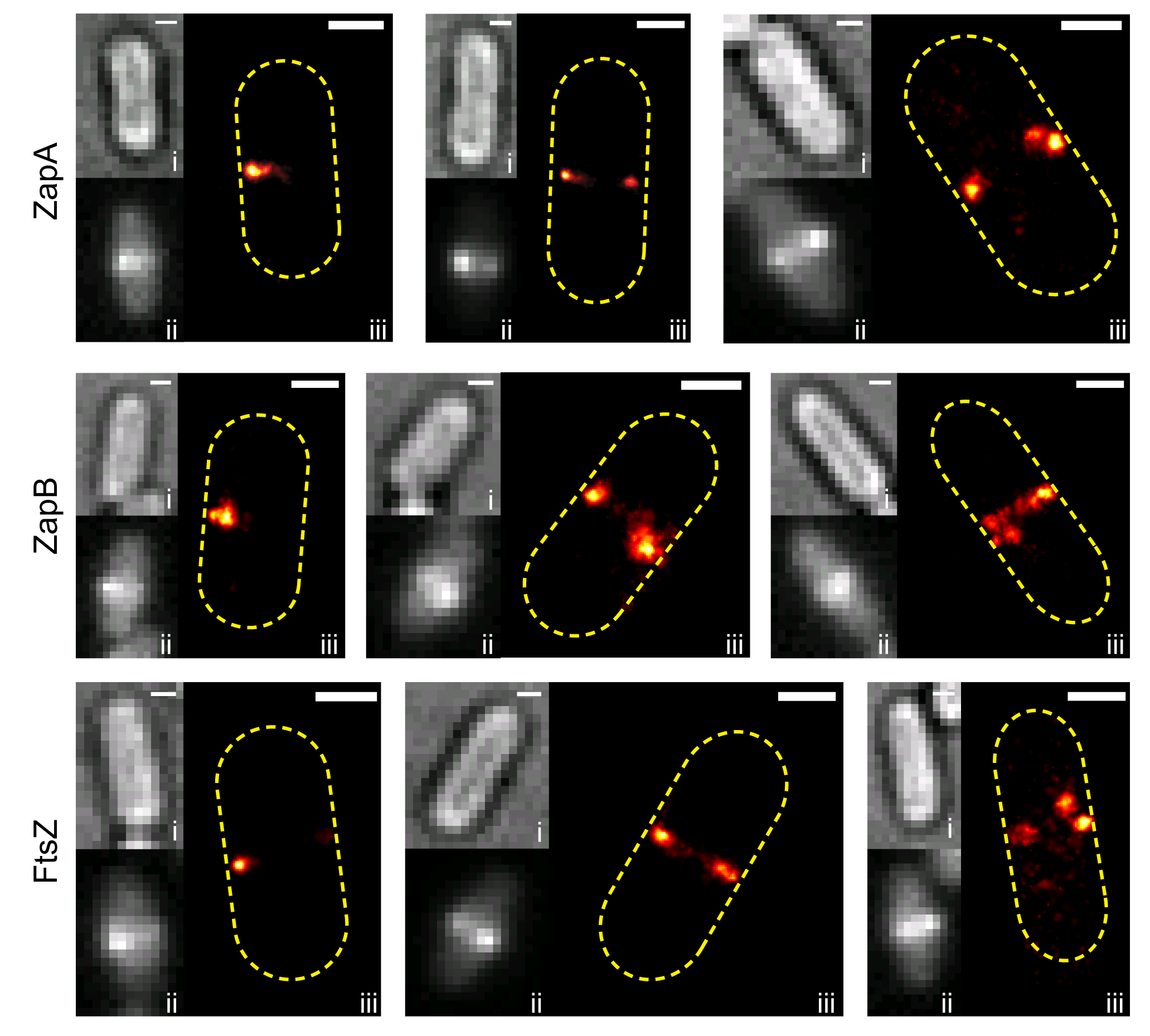

Supplement: S2 Fig — PALM images of mEos2-ZapA (top row), ZapB-mEos2 (middle) and FtsZ-mEos2 (bottom) illustrate the alternate morphologies common to all three protein species – peripheral focus (left), peripheral foci pair (middle), and multiple non-planar structures (right). All images are displayed in order of bright-field (i), ensemble fluorescence (ii), and PALM image (iii). Scale Bars, 500 nm. (TIF) [file pgen.1005128.s003.tif]

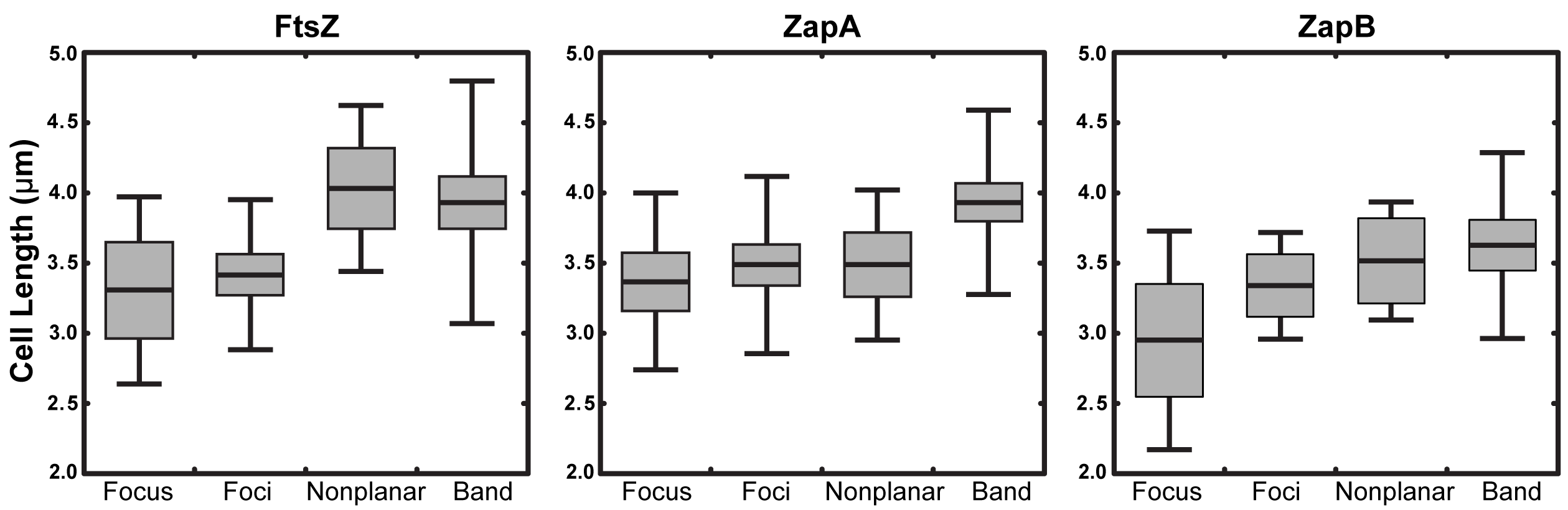

Supplement: S3 Fig — The PALM images for each protein species are categorized according to the four observed structural morphologies and the cell length distribution for each category is plotted as the mean boxed by the 95% confidence interval bounded by the standard deviation. Overlap of two boxed regions indicates the two are not significantly different (p > 0.05). (TIF) [file pgen.1005128.s004.tif]

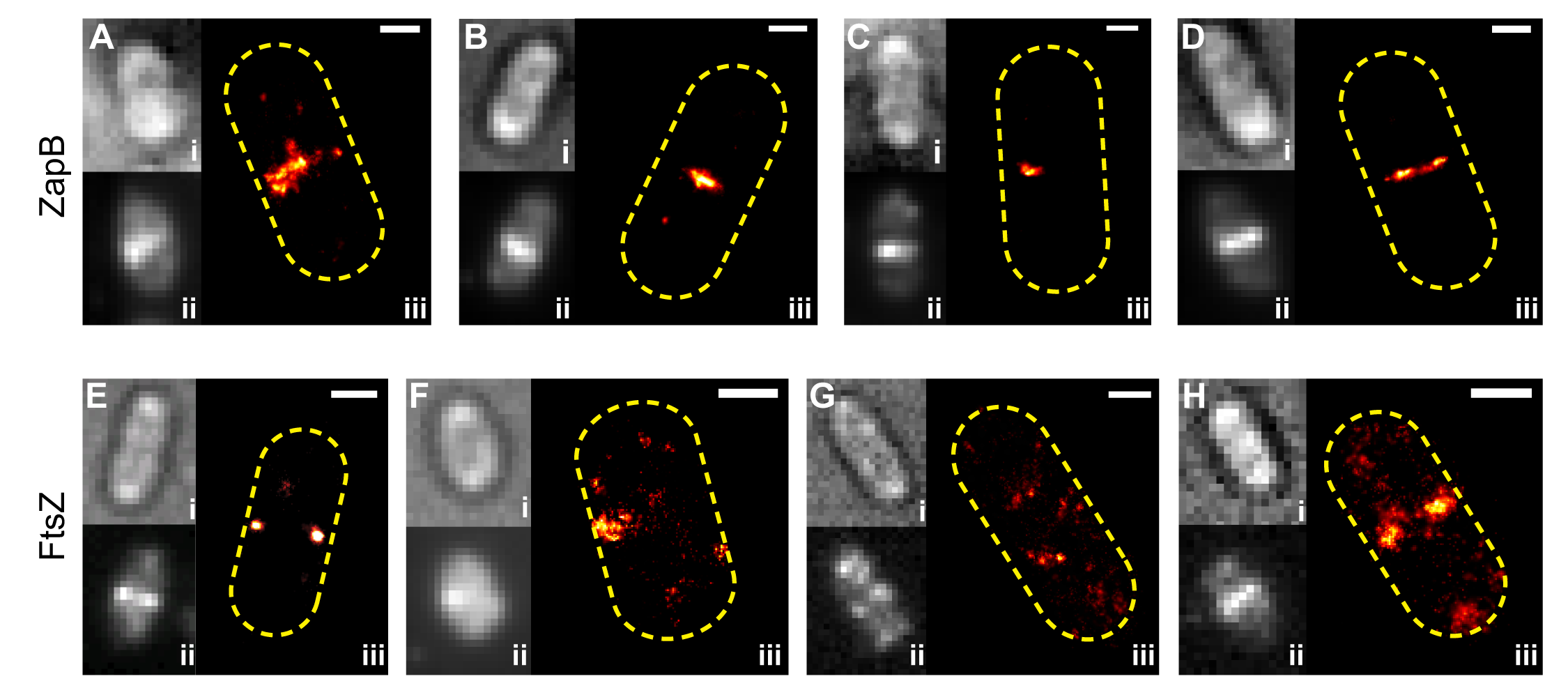

Supplement: S4 Fig — Wt cells labeled with α-ZapB (A-D) or α-FtsZ (E-H) and stained with an Alexa Fluor 647-conjugated secondary antibody (Life Technologies, Inc.) were imaged as described previously [33]. Images are displayed in the order of bright-field (i), ensemble fluorescence (ii) and superresolution image displayed in pseudocolor (iii). Approximate cell outlines are indicated by yellow dashed lines. Scale Bars, 500 nm. (TIF) [file pgen.1005128.s005.tif]

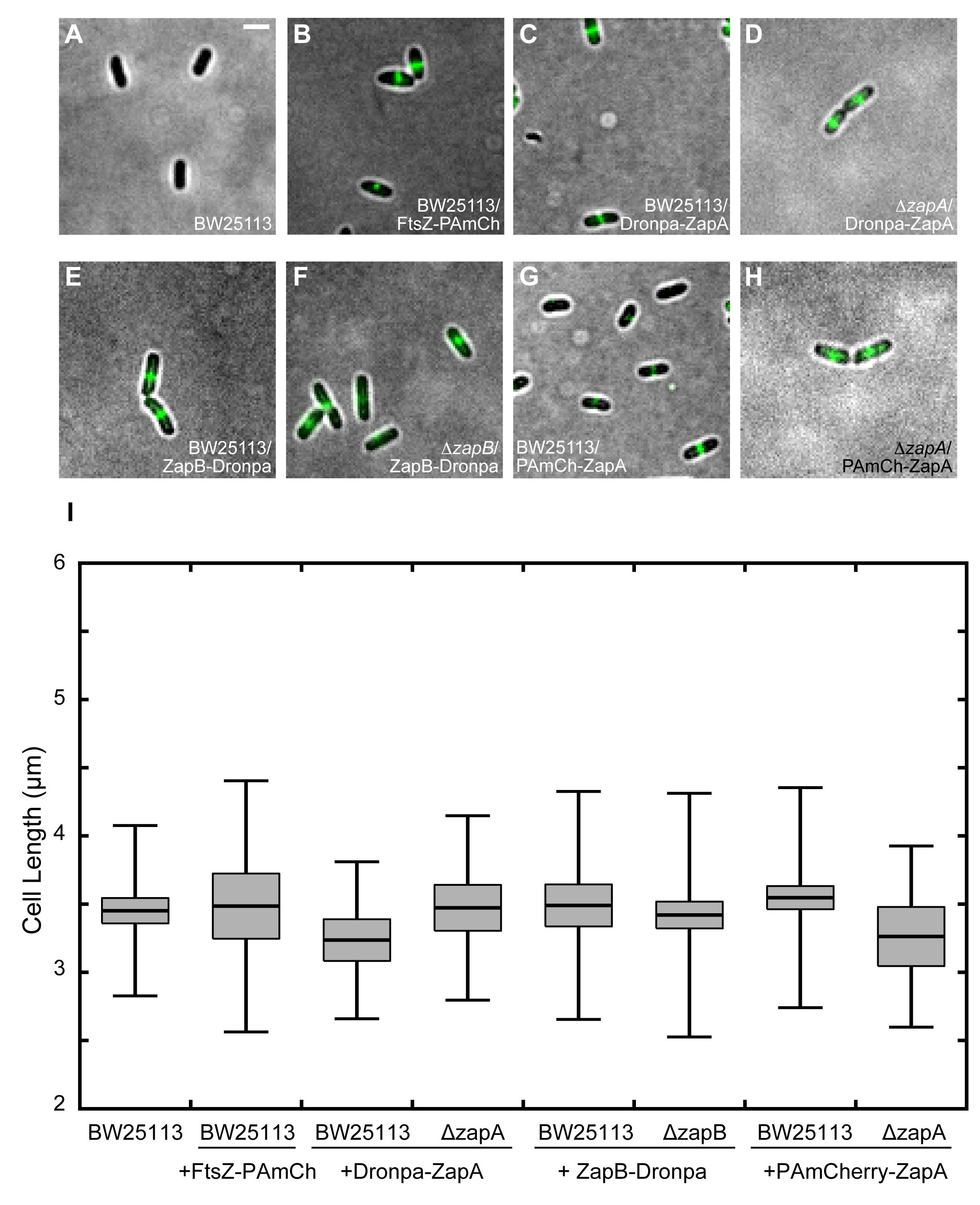

Supplement: S5 Fig — Representative snapshots for wt (A-C,E,G) or deletion strains (D,F,H) expressing FtsZ-PAmCherry1 (B), Dronpa-ZapA (C-D), ZapB-Dronpa (E-F) or PAmCherry1-ZapA (G-H) under slow growth conditions. All images are displayed as ensemble fluorescence images overlaid with bright-field images. (I) Box plots representing cell length distributions are presented as the mean length boxed by the 95% confidence interval bounded by the standard deviation. Scale Bar, 2 μm. (TIF) [file pgen.1005128.s006.tif]

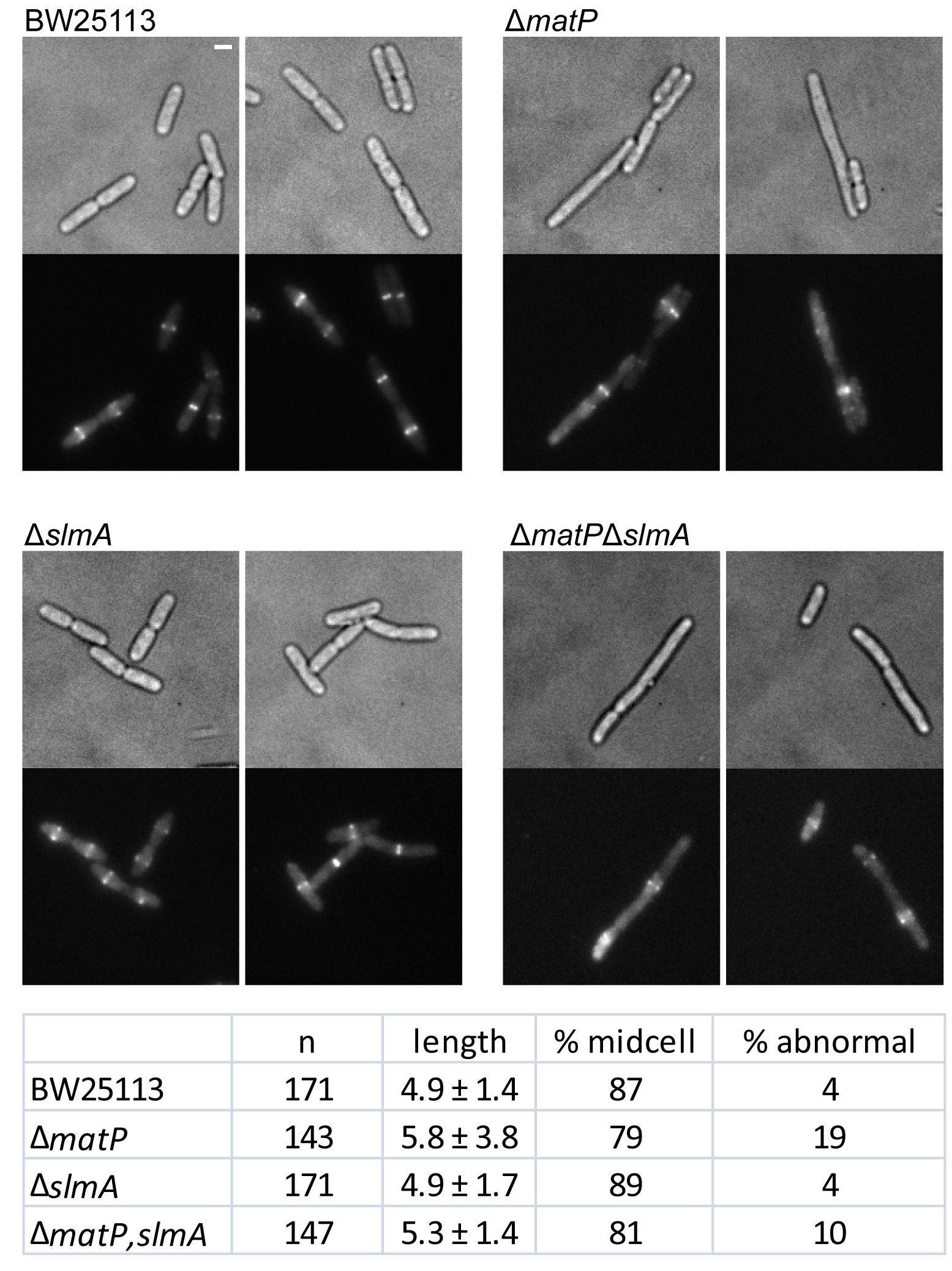

Supplement: S6 Fig — Top: Bright-field and ensemble fluorescence images of wt, ΔmatP, ΔslmA, and ΔmatPΔslmA cells expressing FtsZ-GFP under fast growth conditions. Similar FtsZ mislocalizations were observed both in ΔmatP and ΔmatPΔslmA strains. Bottom: Table displaying cell length and percent of cells displaying abnormal FtsZ localization for all four strains. (TIF) [file pgen.1005128.s007.tif]

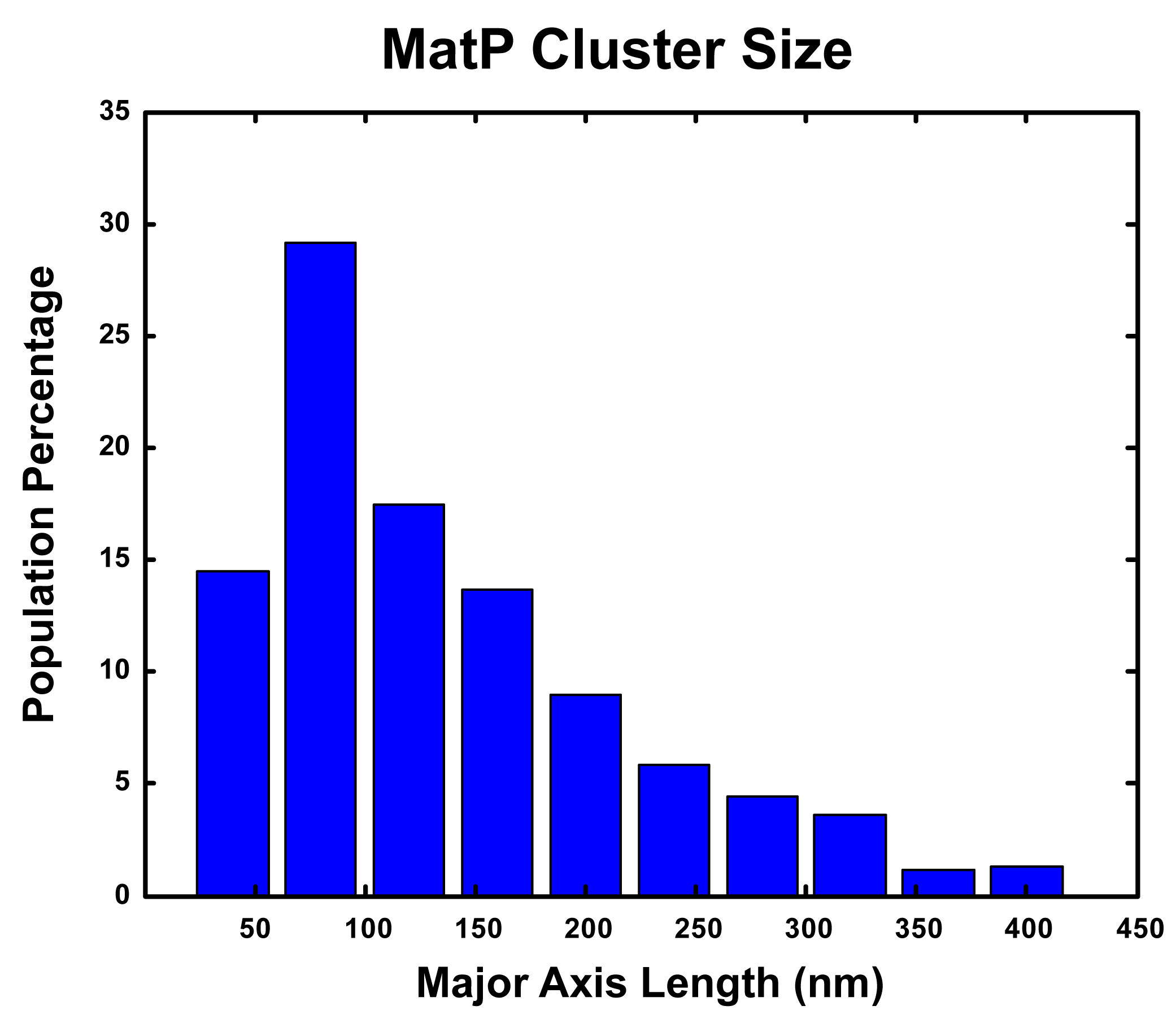

Supplement: S7 Fig — A threshold-based cluster analysis [14] was applied to PALM images of wt or ΔmatP cells expressing MatP-mEos2. We determined the major axis length for each cluster and plotted the distribution as a histogram (blue bars). We found that the average major axis length of MatP-mEos2 clusters was 100 ± 58 nm (x- ± sd). Given that the average eccentricity of MatP-mEos2 clusters was 0.8 (near circular), this measurement represents an estimation of MatP-mEos2 cluster diameter. (TIF) [file pgen.1005128.s008.tif]

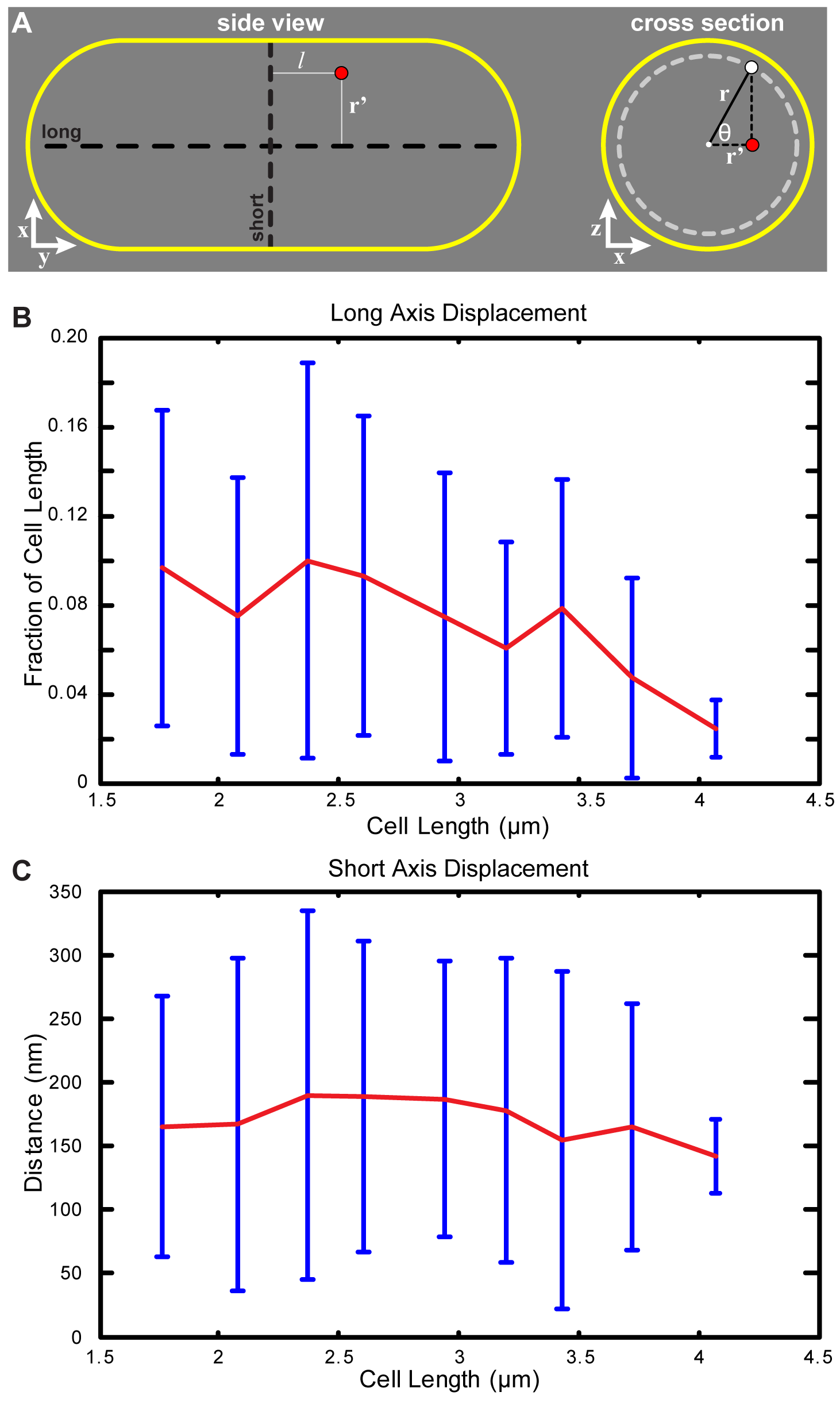

Supplement: S8 Fig — (A) Schematic of measurement geometry. We measured the distance of each MatP-mEos2 cluster (red circle) from the middle of the cell along the short (r’) and long (l) cellular axes (dashed lines). We binned these distances according to cell length and found that MatP clusters undergo a cell-length-dependent migration towards the middle of the cell along the long axis (B). We also found that MatP-mEos2 clusters remain tightly distributed along the short axis with an average displacement of 180 nm (C). Since PALM images are two-dimensional projections of three-dimensional objects (A), the average measured displacement (r') is related to the true radial displacement (r) as described in the S1 Text. (TIF) [file pgen.1005128.s009.tif]

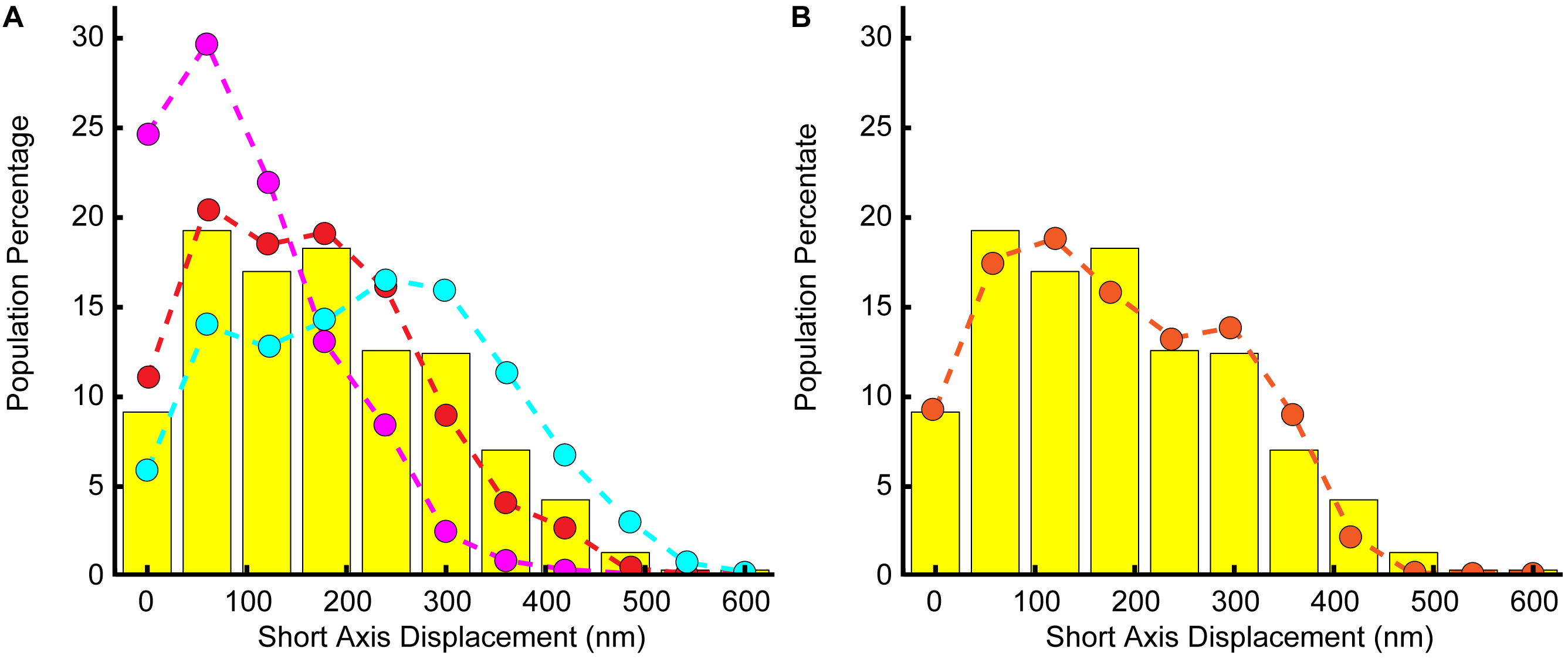

Supplement: S9 Fig — The distribution of distances (r’) separating MatP-mEos2 clusters from the middle of the cell along the short axis is plotted in 60 nm bins (yellow bars, n = 613; see S1 Text). (A) Short axis displacement distributions from three simulations using a Gaussian-distributed radius (r ± sd) and a randomly distributed projection angle (θ) are shown as dotted lines (n = 1000). These simulations were generated with the following parameters: r = 150 ± 100 nm (magenta), 250 ± 100 nm (red), or 350 ± 100 nm (cyan). We found that the radial distribution of MatP-mEos2 clusters was best fit with a true radius of 280 ± 120 nm. (B) The experimental data (yellow) was also well fit by an alternate model (orange) that assumed MatP could uniformly sample the cross-section of a nucleoid with a maximum radius of r max. Least squared fitting found the best-fit r max to be 419 nm, resulting in a distribution defined by 279 ± 97 nm (r ± sd). (TIF) [file pgen.1005128.s010.tif]

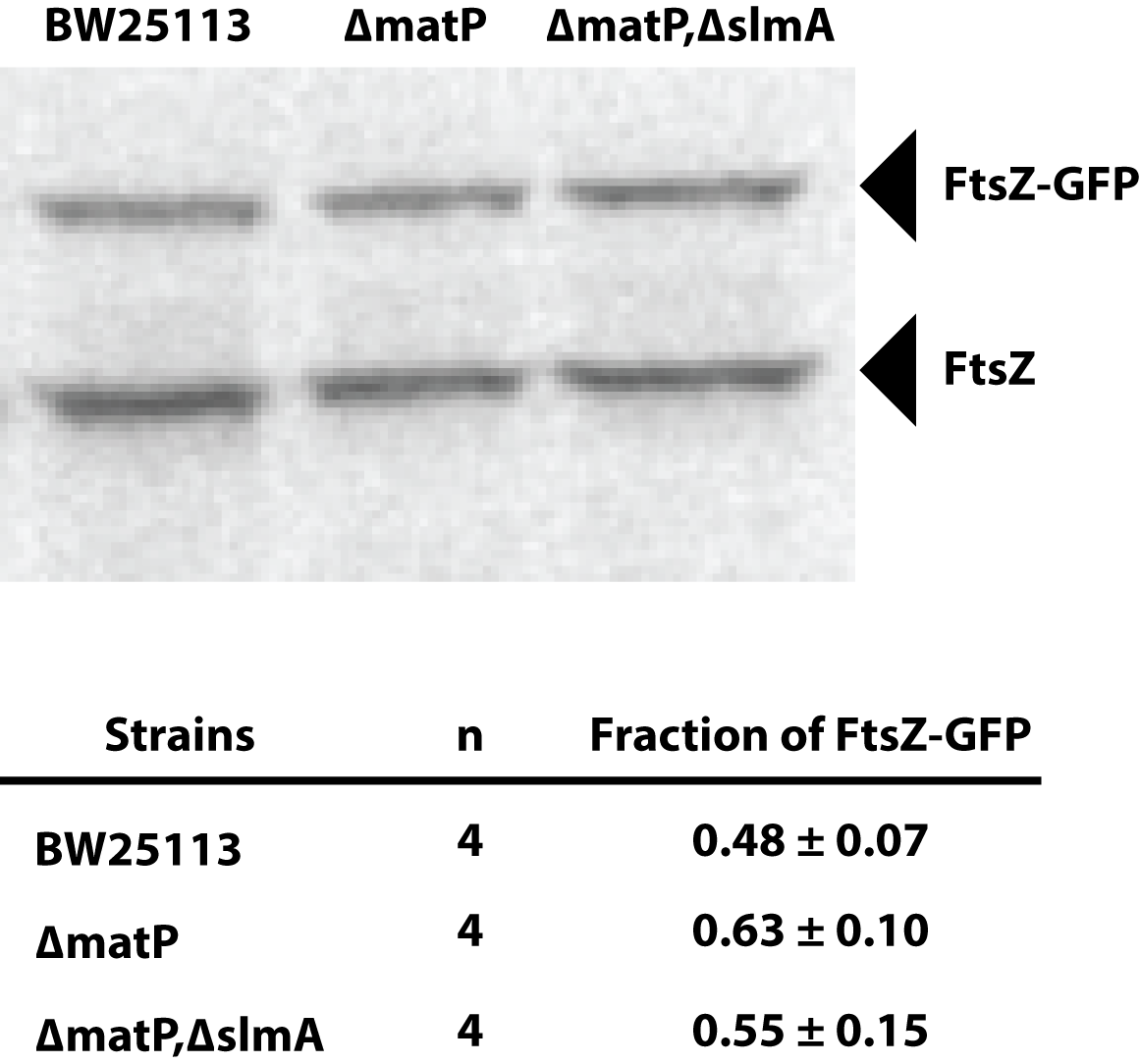

Supplement: S10 Fig — A representative immunoblot of BW25113, ΔmatP, ΔmatPΔslmA cells expressing FtsZ-GFP (pJW0093) stained with α-FtsZ. Signals from FtsZ-GFP and FtsZwt were quantified and used to calculate the fraction of FtsZ-GFP relative to that of FtsZwt. A table summarizing the quantifications is displayed at the bottom. (TIF) [file pgen.1005128.s011.tif]

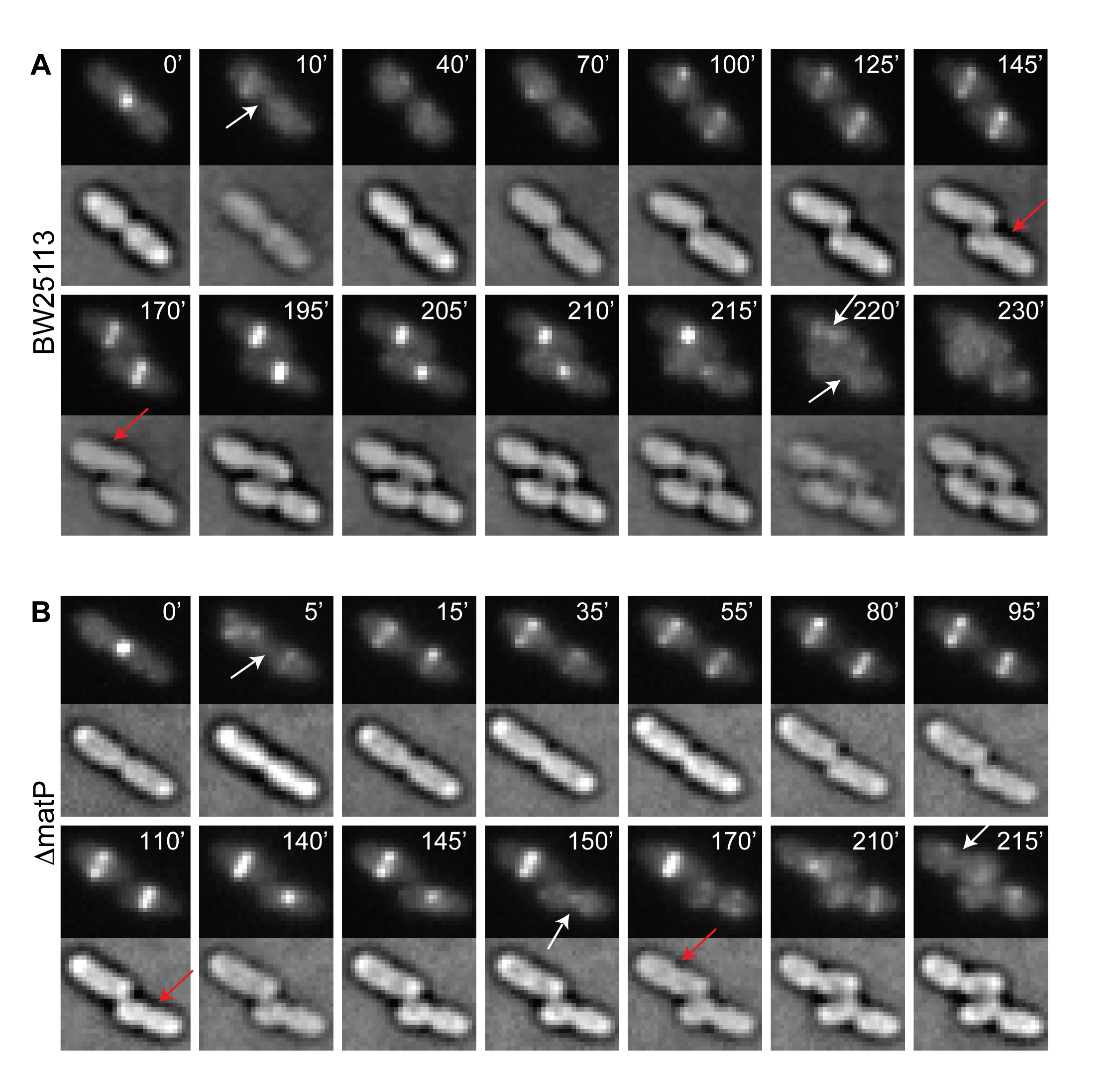

Supplement: S11 Fig — Fluorescence and bright-field images of wt BW25113 (A) and ΔmatP (B) cells expressing FtsZ-GFP (pJW003). Images are displayed as a time-lapse montage with the time-stamp of each image pair indicated at the top right in minutes. White arrows indicate loss of fluorescence from midcell (i.e. beginning/end of cell cycle). Red arrows indicate initiation of cell wall constriction. (TIF) [file pgen.1005128.s012.tif]

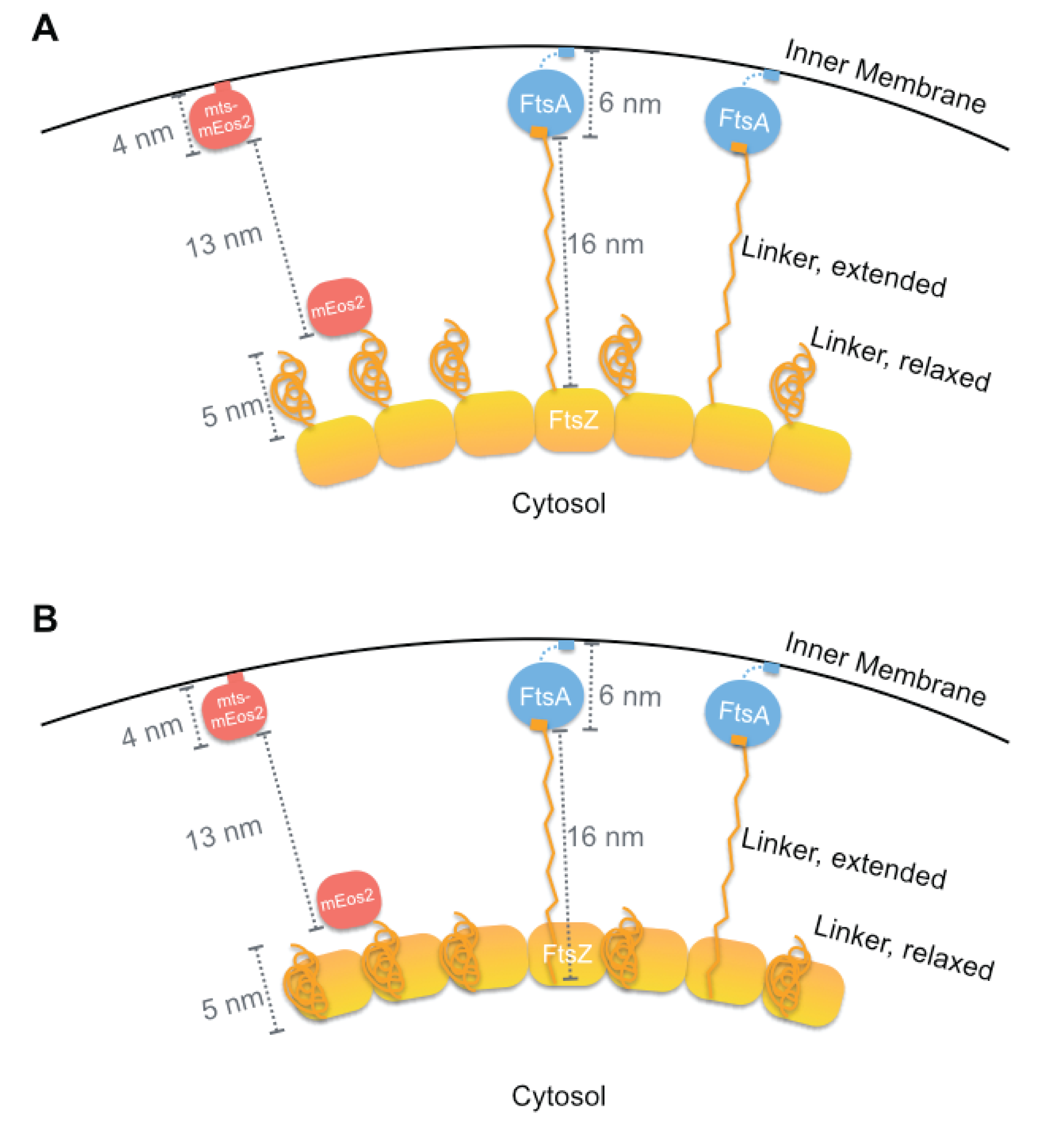

Supplement: S12 Fig — (A) The configuration of the FtsZ linker in the stretched (orange lines) or relaxed (orange coils) state when the C-terminal side (light shade) of FtsZ globular domain (yellow boxes) faces the membrane (see S1 Text). (B) The FtsZ linker configurations when the C-terminal side of FtsZ globular domain faces cytosol according to a recent model [66]. mEos2 (red) and membrane attached FtsA (blue) are drawn to scale. Corresponding distance estimates are labeled individually. (TIF) [file pgen.1005128.s013.tif]
