# Supplementary material for: A Multi-layered Protein Network Stabilizes the Escherichia coli FtsZ-ring and Modulates Constriction Dynamics
Source: PLoS Genet. 2015 Apr 7;11(4):e1005128. doi: 10.1371/journal.pgen.1005128 (PMC4388696; doi:10.1371/journal.pgen.1005128)
Supplement: S1 Table — All PALM images for FtsZ-mEos2, mEos2-ZapA, and ZapB-mEos2 were grouped according to their qualitative appearance. Representative images for non-band structures (i.e. focus, foci and helix) are displayed in S2 Fig, while band structures of non-constricting cells only are shown in Fig 1. (DOCX) [file pgen.1005128.s014.docx]

| **Table S1 PALM Morphology Frequency** | | |  |  |  |
| --- | --- | --- | --- | --- | --- |
|  | n | Focus | Foci | Helix | Band |
| FtsZ-mEos2 | 201 | 0.12 | 0.29 | 0.08 | 0.51 |
| mEos2-ZapA | 229 | 0.16 | 0.33 | 0.09 | 0.41 |
| ZapB-mEos2 | 137 | 0.20 | 0.14 | 0.07 | 0.59 |
