# Supplementary material for: A Multi-layered Protein Network Stabilizes the Escherichia coli FtsZ-ring and Modulates Constriction Dynamics
Source: PLoS Genet. 2015 Apr 7;11(4):e1005128. doi: 10.1371/journal.pgen.1005128 (PMC4388696; doi:10.1371/journal.pgen.1005128)
Supplement: S2 Table — A list of strains and plasmids used in this study. (DOCX) [file pgen.1005128.s015.docx]

| **Table S2 Strains and Plasmids** | |  |
| --- | --- | --- |
| Strain or Plasmid | Relevant genotype | Reference/source |
| BW25113 | K-12, BD792 derivative | Datsenko *et al.* (2000) |
| JW2878 (Keio) | BW25113, *zapA*::kan | Baba *et al.* (2006) |
| JW3899 (Keio) | BW25113, *zapB*::kan | Baba *et al.* (2006) |
| JW0939 (Keio) | BW25113, *matP*::kan | Baba *et al.* (2006) |
| JB636 | BW25113, *matP*::frt, *slmA*::kan | This study |
|  |  |  |
| pJB051 | ColEI, P_T5-lac_::mEos2-ZapA, *cat* | This study |
| pJB045 | ColEI, P_T5-lac_::ZapB-mEos2^1^, *cat* | This study |
| pJB091 | ColEI, P_T5-lac_::ZapB-mEos2^2^, *cat* | This study |
| pJB042 | ColEI, P_T5-lac_::FtsZ-mEos2, *cat* | Buss *et al.* (2013) |
| pJB057 | ColEI, P_T5-lac_::Dronpa-ZapA, *cat* | This study |
| pJB073 | ColEI, P_T5-lac_::ZapB-Dronpa, *cat* | This study |
| pJB058 | ColEI, P_T5-lac_::6xHis-FtsZ-PAmCherry1, *cat* | This study |
| pJB056 | pSC101, P_BAD_::*zapA*, *aadA* | Buss *et al.* (2013) |
| pJB066 | pSC101, P_BAD_::FtsZ-PAmCherry1, *aadA* | This study |
| pJB089 | ColEI, P_T5-lac_::Dronpa-ZapA—FtsZ-PAmCherry1, *cat* | This study |
| pJB061 | pSC101, P_BAD_::PAmCherry1-ZapA, *aadA* | This study |
| pJB090 | ColEI, P_T5-lac_::Dronpa-ZapA—PAmCherry1-ZapA, *cat* | This study |
| pXY029 | ColEI, P_T5-lac_::mEos2-MTS_Bs_, *cat* | This study |
| pXY027 | ColEI, P_T5-lac_::FtsZ-GFP, *cat* | This study |
| pJB043 | pMB1, P_Lac_::FtsZ-mEos2, *bla* | This study |
| pJB128 | pMB1, P_Lac_::MatP-mEos2, *bla* | This study |
| pJB154 | ColEI, P_T5-lac_::GFP-ZapA, *cat* | This study |
| pJB150 | ColEI, P_T5-lac_::GFP-ZapB, *cat* | This study |
| JW0093 | ColEI, P_T5-lac_::6xHis-FtsZ-GFP, *cat* | Kitagawa *et al.* (2005) |
