# Supplementary material for: A Multi-layered Protein Network Stabilizes the Escherichia coli FtsZ-ring and Modulates Constriction Dynamics
Source: PLoS Genet. 2015 Apr 7;11(4):e1005128. doi: 10.1371/journal.pgen.1005128 (PMC4388696; doi:10.1371/journal.pgen.1005128)
Supplement: S3 Table — A list of primers used in this study. (DOCX) [file pgen.1005128.s016.docx]

| Table S3 Primer List | |  |
| --- | --- | --- |
| 1 | SpeI-mEos2-5F | AATTACTAGTATGAGTGCGATTAAGCCAGA |
| 2 | mEos2-Link-3R | AATTGAATTCGCCAGAACCAGCAGCGGAGCCAGCGGATCCTCGTCTGGCATTGTCAGG |
| 3 | EcoRI-ZapA-5f | ATATGAATTCATGAGTGCGATTAAGCCAGA |
| 4 | ZapA-NotI-3R | ATATGCGGCCGCTCATTCAAAGTTTTGGTTAGTTTTTTC |
| 5 | SpeI-ZapB-5F | ATATACTAGTATGACAATGTCATTAGAAGTGTTTGA |
| 6 | ZapB-Link-3R | TTAAGAATTCGCCAGAACCAGCAGCGGAGCCAGCGGATCCGACCTCTTCCATGCGAC |
| 7 | EcoRI-mEos2-5f | ATATGAATTCATGAGTGCGATTAAGCCAGA |
| 8 | mEos2-NotI-3R | TTAAGCGGCCGCTTAATCAGCTTGCTTACGCAG |
| 9 | ZapB-SfoI-3R | TTTAAAGGCGCCGGGGATGCTGACCTCTTCCATGCGAC |
| 10 | SfoI-mEos2-5f | AAATTTGGCGCCATGAGTGCGATTAAGCCAGACAT |
| 11 | SpeI-Dronpa-5F | ATATACTAGTATGGTGAGTGTGATTAAACCAGACAT |
| 12 | Dronpa-Link-3R | TATAGCTAGCGCCAGAACCAGCAGCGGAGCCAGCGGATCCCTTGGCCTGCCTCGGCAGCT |
| 13 | NheI-ZapA-5f | AAATGCTAGCATGTCTGCACAACCCGTCGA |
| 14 | SfoI-Dronpa-5f | AAATTTGGCGCCATGGTGAGTGTGATTAAACCA |
| 15 | Dronpa-NotI-3R | TAATGCGGCCGCTTACTTGGCCTGCCTCGGCA |
| 16 | NotI-mCherry-5f | TTTAGCGGCCGCATGGTGAGCAAGGGCGAG |
| 17 | mCherry-NotI-3R | AAATGCGGCCGCTTACTTGTACAGCTCGTCCAT |
| 18 | NheI-FtsZ-5f | TTTAGCTAGCATGTTTGAACCAATGGAACTTA |
| 19 | mCherry-SalI-3R | AATTGTCGACTTACTTGTACAGCTCGTCCATGCCGCCG |
| 20 | HindIII-RBS-5F | AATTAAGCTTTAACTTTAAGAAGGAGATATACGCTAGCATG |
| 21 | mCherry-HindIII-3R | AATTAAGCTTTTACTTGTACAGCTCGTCCATGCCG |
| 22 | SpeI-Dronpa-5F | ATATACTAGTATGGTGAGTGTGATTAAACCAGACAT |
| 23 | NheI-mCherry-5f | TTAAGCTAGCATGGTGAGCAAGGGCGAGG |
| 24 | PAmChery-Link-3R | TATAGCTAGCGCCAGAACCAGCAGCGGAGCCAGCGGATCCCTTGTACAGCTCGTCCATGC |
| 25 | ZapA-HindIII-3R | AATTAAGCTTATGTCTGCACAACCCGTCGATATC |
| 26 | MTS-3R | TTAATCTTAGCCATCATTCCTTTGTTTTGCTCTTCAAGCACTCGTCTGGCATTGTCAGGC |
| 27 | NotI-MTS-3R | ATTGCGGCCGCTTAAGATCTTACTCCGAAAAATGACTTAATCTTAGCCATCATTCCTTTGTTTTGC |
| 28 | GFP-NotI-3R | AAATGCGGCCGCTTATTTGTATAGTTCATCCATGCCA |
| 29 | XbaI-FtsZ-5f | ATTATCTAGATTTAAGAAGGAGATATACATATGTTTGAACCAATGGAACTTAC |
| 30 | SnaBI-mEos2-3r | TTAATACGTATTATCGTCTGGCATTGTCAGGCAAT |
| 31 | XbaI-MatP-5f | ATTATCTAGATTTAAGAAGGAGATATACATATGAAATATCAACAACTTGAAAATCTTG |
| 32 | MatP-BamHI-3R | ATAGGATCCTTCCTTACCCAGCAATGCCTGC |
| 33 | SpeI-GFP-5F | ATAACTAGTATGAGTAAAGGAGAAGAACTTTTCACT |
| 34 | GFP-NheI-3r | ATAGCTAGCTTTGTATAGTTCATCCATGCCATGTGT |
| 35 | NheI-ZapB-5f | AAATTGCTAGCGCTGGCTCCGCTGCTGGTTCTAGCGGCCGCACAATGTCATTAGAAGTGTTTGAG |
